# Identification of a novel deeply quiescent neural stem cell population in the subventricular zone: a potential source for brain repair

**DOI:** 10.1038/s41421-026-00914-4

**Published:** 2026-07-14

**Authors:** Arup R. Nath, Sanskar Ranglani, Mohd Yaseen Malik, Jacek Szymanski, Anis Uz Zaman, Emmanouela Repapi, Roy Drissen, Natalie M. Doig, Peter J. Magill, Claus Nerlov, Liliana Minichiello

**Affiliations:** 1 Department of Pharmacology, University of Oxford, Oxford, UK; 2 MRC Unit, Weatherall Institute of Molecular Medicine, University of Oxford, John Radcliffe Hospital, Oxford, UK; 3 MRC Molecular Haematology Unit, Weatherall Institute of Molecular Medicine, University of Oxford, John Radcliffe Hospital, Oxford, UK; 4 Brain Network Dynamics Unit, Nuffield Department of Clinical Neurosciences, University of Oxford, Oxford, UK; 5 Medical Research Council Centre of Research Excellence in Restorative Neural Dynamics, Oxford, UK

**Keywords:** Stem cells, Neural stem cells

Dear Editor,

Canonically, adult neurogenesis occurs in two regions of the mammalian brain, the ventricular-subventricular zone (V-SVZ) and the subgranular zone, and modulates associated brain circuitry^1^. The V-SVZ lining the walls of the lateral ventricles harbors the largest neurogenic niche in mammals^2^. In rodents, quiescent neural stem cells (qNSCs) in the V-SVZ give rise to transit-amplifying cells, which differentiate into neuroblasts and further migrate to the olfactory bulb to produce interneurons^3^. Interestingly, in adult humans, the neurogenic niche of the V-SVZ can produce new neurons that integrate into the striatum^4,5^. The ability to modulate the generation and integration of new neurons in the adult brain holds the promise of treating several neurodegenerative diseases. Therefore, it has been the focus of intensive research over the last few decades^6^.

A prerequisite for such modulation is a comprehensive and unbiased understanding of the molecular and cellular heterogeneity of these cells. Several advances have been achieved in this field. Numerous studies have used single-cell RNA sequencing (scRNA-seq) to identify and characterize the continuum of neurogenesis that defines the murine V-SVZ^7–9^, dissecting the transcriptional identity and heterogeneity of these cells at unprecedented resolution. However, these studies relied on either a reporter line or specific antibodies targeting canonical markers of known V-SVZ populations (PROM1, GLAST, GFAP, EGFR) to isolate various cell types and states, which may have limited the identification of potentially relevant new cell types.

Indeed, by integrating datasets from six studies that previously sequenced the V-SVZ, Apostolou and Donega^10^ confirmed that using different reporter lines and/or enriching for markers of interest favored certain NSC states over others. This is further evidenced by the finding that, while deeply qNSCs express prolactin receptor (PRLR) and serotonergic receptor (5-HT2C), which are vital for their function^11,12^, cells expressing these markers have not been isolated or sequenced. Therefore, it is reasonable to presume that reporter lines or antibodies used to isolate known cellular populations may have prevented the discovery of such cells, which may originate from a distinct subset of qNSCs compared to the canonically accepted GLAST^+^ qNSCs. Here, by combining a novel reporter line, the prepro-enkephalin *(Penk)-Cre*^*Ai9*^ line, which labels enkephalinergic lineage-derived cells within the V-SVZ, with scRNA-seq, we identified a previously unrecognized population of deeply qNSCs in the V-SVZ that expresses *Htr2c* and *Prlr,* and provided compelling evidence that these cells are more quiescent, transcriptionally distinct from the previously characterized GLAST^+^ qNSCs and with a conserved signature across species.

First, we showed that the Penk-lineage-derived tdTomato^+^ cells on the V-SVZ wall, in contrast to those in the striatal parenchyma, lacked enkephalin (ENK) expression, suggesting that these cells expressed ENK at some point during their genesis but have since ceased to express it. The morphological features resembled those observed in electron micrographs of qNSCs, a specialized type of astrocyte, also known as type B1 cells in the V-SVZ niche^12^, especially the bipolar projections running parallel to the lateral wall of the lateral ventricle (Supplementary Fig. S1a–c). Further characterization using canonical V-SVZ neurogenic niche markers revealed that V-SVZ tdTomato^+^ cells from the *Penk-Cre*^*Ai9*^ line are neurogenic (Supplementary Fig. S1d–p). Moreover, we identified tdTomato expression in the striato-pallidal neural epithelium of the *Penk-Cre*^*Ai9*^ line from embryonic day 11.5 (E11.5) onwards, consistent with reports of NSCs identified as early as E11.5 (Supplementary Fig. S2a–f). And we confirmed that some tdTomato^+^ cells from the V-SVZ migrate along the rostral migratory stream to give rise to different classes of interneurons in the olfactory bulb (Supplementary Fig. S3a–n), supporting the conclusion that of the *Penk-Cre*^Ai9^ line reliably labels some qNSCs and cells further along the differentiation trajectory of the V-SVZ neurogenic niche.

To fully characterize and identify the transcriptional profile of the newly discovered tdTomato^+^ cells in the V-SVZ of the *Penk-Cre*^*Ai9*^ line, we performed fluorescence-activated cell sorting based on a refined sorting strategy combining tdTomato fluorescence with the cell surface antigen, CD11b, followed by scRNA-seq of the adult V-SVZ/striatal resected tissue (Supplementary Fig. S4a). Interestingly, in this dataset (referred to as “dataset A”), we identified three cell clusters via hierarchical clustering: two qNSC clusters (B cells) and one migrating neuroblast cluster (Supplementary Fig. S4b). Using differential gene expression analysis, we examined each cluster’s unique markers to determine the clusters’ identities. The newly identified cluster 1 (henceforth referred to as B0 cells) was characterized by a set of genes previously shown to play pivotal roles in qNSC maintenance and function. These included Klotho (*KI*), a gene vital for NSC maintenance; Transthyretin (*Ttr*), a thyroid hormone transporter; Sclerostin domain-containing protein 1 (*Sostdc1*), uniquely expressed by B0 cells; and Retinol dehydrogenase 5 (*Rdh5*). Other genes expressed exclusively in B0 cells or cluster 1 included chloride and potassium channels, such as *Clic6, Kcne2*, and *Kcnj13*, which had not previously been linked to NSC function (Supplementary Fig. S4c). The other two clusters identified were consistent with previously known populations within the V-SVZ NSC niche. Cluster 2 (henceforth called B1 cells) showed numerous previously characterized canonical markers of V-SVZ qNSCs, such as connexins (*Gja1*, *Gjb6*), *Etnppl*, and *Itih3*^8^. Finally, cluster 3 (migrating neuroblasts) was characterized by well-known markers, such as *Sox11*, *Gad1*, and *Tubb3* (Supplementary Fig. S4c).

Having isolated two distinct B cell clusters in dataset A, we examined their transcriptional differences. Using DESeq2, we identified 720 differentially expressed genes between B0 and B1 cells, with a false discovery rate < 0.05. Key among them was *Slc1a3* (GLAST), which was highly upregulated exclusively in B1 cells and virtually absent in B0 cells (Supplementary Fig. S4d and Table S1).

GLAST^+^ qNSCs have long been considered the most quiescent NSC population in the V-SVZ, and this understanding has influenced the design of many scRNA-seq experiments^8^. However, given its virtual absence in B0 cells, we delved deeper into their transcriptional profile. Thus, we conducted pathway analysis to better understand the newly identified qNSC cluster (B0). Gene ontology analysis revealed greater upregulation of energy metabolism-related pathways, particularly mitochondrial-dependent pathways such as ATP synthesis and the electron transport chain, in B0 cells than in B1 cells (Supplementary Fig. S4e). This profile aligns with reports that qNSCs exhibit a metabolically active state that relies on high energy consumption via mitochondrial oxidative phosphorylation to maintain quiescence. In contrast, GLAST^+^ B1 cells showed enrichment in multiple pathways associated with neuronal differentiation, such as synapse assembly, regulation of synapse organisation, postsynaptic density, postsynaptic membrane, cognition, and neuron projection (Supplementary Fig. S4e). These results suggest that the canonically accepted GLAST^+^ qNSCs may not represent the most quiescent NSCs population in the V-SVZ, and that a more quiescent, less differentiated NSCs population may be marked by other genes, such as those expressed in B0 cells, as shown in this study. We also confirmed consistent isolation of these neurogenic cells using an additional sorting strategy to collect tdTomato^+^ cells (Supplementary Fig. S5a–f).

To investigate whether B0 cells preceded B1 cells in their differentiation trajectory, we conducted pseudotime analysis with Slingshot, which revealed a single continuous trajectory from B0 cells to migrating neuroblasts (Fig. 1a). Next, to contextualize our findings within the existing literature, we integrated our dataset with a previously published scRNA-seq study of the murine V-SVZ neurogenic niche by Dulken et al.^8^ We used the standard Seurat pipeline to identify mutual nearest neighbours based on the expression of the top 2000 genes, enabling a direct comparison between the two datasets. While B1 cells and migrating neuroblasts clustered near the qNSC populations and neural progenitor cell (NPC)-like cells, respectively, as identified by Dulken et al., strikingly, B0 cells identified in this study formed a distinct cluster, separate from all cell types in the Dulken et al.^8^ dataset (Fig. 1b), indicating a previously uncharacterized cellular state.

**Fig. 1:**
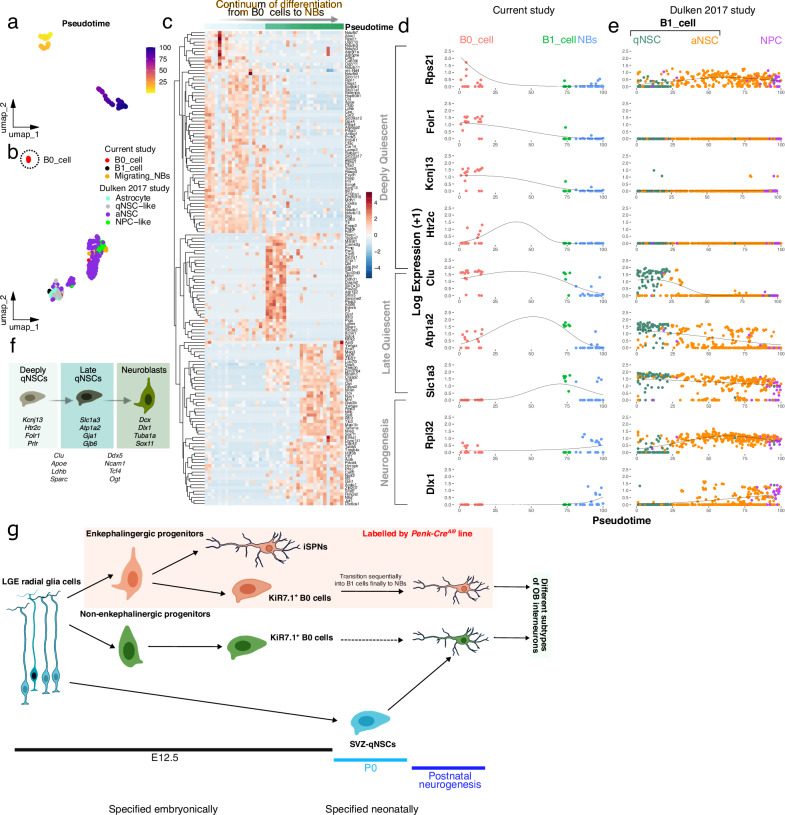
B0 cells are less differentiated and more quiescent than the previously characterized qNSCs from the murine V-SVZ. **a** FeaturePlot of pseudotime values for this study’s dataset A. **b** UMAP plot from integrating this study’s dataset A with the Dulken et al. dataset. B0 cells: deeply quiescent neural stem cells of the V-SVZ; B1 cells: late quiescent neural stem cells of the V-SVZ; NBs: neuroblasts; qNSC: quiescent neural stem cell; aNSC: activated neural stem cell; NPC: neural progenitor cell. **c** Heatmap of scaled gene expression for the top 150 genes associated with pseudotime in our dataset A, identified using a generalized additive model. The top arrow indicates the differentiation continuum from panel **a**. **d**, **e** Scatterplots showing log expression (+1) values of key genes across pseudotime in this study’s dataset A (**d**) versus Dulken et al. dataset (**e**). Deeply quiescent genes: *Rps21*, *Folr1*, *Kcnj13*, *Htr2c*; late quiescent genes: *Clu*, *Atp1a2*, *Slc1a3*; and neurogenesis genes: *Rpl32*, *Dlx1*. **f** Graphic showing the cell types isolated in our dataset A and their unique and shared gene markers. **g** The proposed model suggests multiple origins of adult V-SVZ neural stem cells. Radial glia in the lateral ganglionic eminence (LGE) give rise to adult canonical V-SVZ NSCs (B1/GFAP^+^ cells) derived from embryonic/neonatal stages, and to adult V-SVZ NSCs (KiR7.1^+^/tdTomato^+^ and KiR7.1^+^/tdTomato^-^ B0 cells) derived from embryonic (E12.5) quiescent progenitors. Upon stimulation, KiR7.1^+^/ tdTomato^+^ B0 cells sequentially transition to B1, NBs, and finally to diverse interneuron types like canonical B1/GFAP^+^ cells (Supplementary Fig. S12a–e). Panels **f** and **g** were created using NIH BioArt.

To identify the specific gene expression dynamics that characterize this developmental progression, we modeled gene expression as a non-linear function of pseudotime using a generalized additive model implemented with the tradeSeq in the fitGAM package^13^. This analysis revealed genes expressed exclusively in each cell type and highlighted markers shared between states or gradually changing between them (Fig. 1c). Therefore, the genes enriched in the earliest pseudotime (represented by B0 cells) included *Prlr*, *Enpp2*, *Kcnj13*, *Htr2c*, *Folr1*, and *Rps21*. Genes enriched in the middle of the trajectory (represented by B1 cells) were previously identified as markers of GLAST^+^ B cells, including *Itih3* and *Gja1*. Notably, we also found that several genes previously identified as markers of qNSCs were co-expressed in both B0 and B1 cells, including *Clu*^14^, *Atp1a2*^8^, and *Apoe*^15^, among others.

To further scrutinize the differences between this study’s datasets and the previously published Dulken et al.^8^ dataset, we performed a similar analysis by directly comparing the expression trajectories of key genes in our dataset with those in the Dulken et al. dataset (Fig. 1d, e). This comparison revealed that genes upregulated at the earliest pseudotime in dataset A, such as *Kcnj13*, *Htr2c*, and *Folr1*, were virtually undetected in the Dulken et al. dataset^8^. Conversely, genes shared between B0 and B1 cells in our dataset correlated with the earliest pseudotime in the Dulken et al. dataset^8^ (*Clu*, *Atp1a2*), as did the genes enriched only in B1 cells in dataset A of this study (*Slc1a3*). Finally, neuronal differentiation markers, such as *Dlx1* and *Rpl32*, were upregulated later in pseudotime in both datasets: this study and Dulken et al.^8^

These findings strongly suggest that the B0 population represents a deeper, more quiescent state that precedes the qNSC state characterized by Dulken et al.^8^ study. Critically, the unique molecular signature of the B0 cells includes genes with established functional roles in NSC quiescence and activation that were absent from the Dulken et al.^8^ transcriptomic dataset. *Htr2c*, for example, encodes a serotonergic receptor that plays a crucial role in qNSC function^11^. Similarly, *Prlr* encodes the prolactin receptor, which is critically involved in NSC regenerative activity^12^. These results confirmed that we isolated a transcriptionally distinct, previously unidentified, deeply qNSC population. While previous studies implied its functional existence, it had, until now, eluded transcriptomic characterization at the single-cell level^7,8^.

We then sought to identify and localize the B0 cell population in the murine V-SVZ using immunostaining. For example, *Kcnj13*, found exclusively in B0 cells, encodes the inwardly rectifying potassium channel KiR7.1. We found that expression of the B0 cell marker KiR7.1 was restricted to the V-SVZ, colocalizing with some tdTomato^+^ cells displaying a stellate morphology characteristic of qNSCs of the V-SVZ (Supplementary Fig. S6a–l). Similar results were obtained for the PRLR (Supplementary Fig. S6p–r) and the B1 cell markers, GFAP (Supplementary Fig. S6m–o) and GLAST (Supplementary Fig. S6s–u).

Since tdTomato in the *Penk-Cre*^*Ai9*^ line labels a range of cell types across different developmental stages within the V-SVZ, including astrocyte-like (qNSCs), we aimed to functionally validate these findings (Supplementary Fig. S7). We found that 5-fluorouracil treatment, an antimitotic drug that selectively reduces proliferating cells while sparing non-proliferating cells, did not alter the number of qNSCs (tdTomato^+^/GFAP^+^ or tdTomato^+^/KiR7.1^+^) in the *Penk-Cre*^*Ai9*^ V-SVZ (Supplementary Fig. S7a–k). In addition, the creation of a partial model of Parkinson’s disease confirmed that some tdTomato^+^ cells in the *Penk-Cre*^*Ai9*^ line are quiescent and can be activated in response to neuronal injury to support striatal tissue repair (Supplementary Fig. S8a–g, h–p). Furthermore, in vivo and in vitro functional modulation of B0 cell markers, including *Htr2c, Prlr, Kl*, and *Ttr*, further supported the neurogenic nature of B0 cells and strengthened our findings (Supplementary Table S2). We also provided evidence that expression levels of B0 cell markers *(Kcnj13, Prlr, Kl*), in contrast to B1 cell markers (*Slc1a3, Id3*), increase with age, supporting a deeper quiescent state (Supplementary Fig. S9a–f).

Finally, we identified B0 cells in the human V-SVZ by reanalysing the single-nucleus RNA-seq dataset from Puvogel et al. (Supplementary Fig. S10a–d) and provided experimental validation of single-cell data by isolating B0 cells using KiR7.1 antibodies (Supplementary Fig. S11).

In summary, the isolation and characterization of qNSCs from the V-SVZ have been at the forefront of regenerative neurobiology, with several advances in this field^10^. Our study uncovers a previously unidentified deep qNSC population that precedes the canonical GLAST^+^ NSC population and encompasses all steps of the differentiation continuum from B0 to neuroblast precursors and, finally, to new olfactory neurons. While multiple key markers of the B0 cluster identified in this study were associated with qNSC function, namely *Prlr* and *Htr2c*^11,12^, studies that previously performed scRNA-seq failed to identify populations with a consistent transcriptional profile. By comparing our data with the Dulken et al. dataset^8^, we demonstrated that this population is distinct from any NSC populations they previously identified. Moreover, shared gene expression patterns, albeit at different levels, are higher in B0 cells. Examples include *Bsg, Cd63, Ndufa13, Ctsd, Slc39a12*, and *Selenos*. However, these genes remain detectable in B1 cells, with a gradual decrease (Fig. 1c). This pattern is consistent with an intermediate transcriptional state that marks progression from deeper to late quiescence within the qNSC pool, suggesting that B0 cells transition to B1 cells. Our proposed model in Fig. 1g, based on the ontogenesis of B0 cells (Supplementary Fig. S12), supports multiple origins of adult V-SVZ NSCs, namely, adult canonical V-SVZ NSCs (B1/GFAP^+^ cells) derived from embryonic/neonatal radial glia and adult V-SVZ NSCs (B0/KiR7.1^+^/tdTomato^+^ and B0/KiR7.1^+^/tdTomato^-^ cells) derived from early (E11.5/E12.5) enkephalinergic or non-enkephalinergic quiescent embryonic progenitors.

Furthermore, in line with reports that qNSCs have sparse gene expression profiles, other studies that isolated a very large number of cells, albeit unbiasedly, at much lower sequencing depths may have been unable to identify this subgroup. This is because standard bioinformatics pipelines favor cells with higher gene expression^10^. By integrating datasets from six different studies, Apostolou and Donega^10^ confirmed that datasets comprising a large number of cells may be ‘too noisy’ to accurately uncover qNSCs. Consistent with this, our dataset’s strength lies in its deep-sequencing approach and analysis of significantly fewer cells than in previous studies, thereby allowing us to examine these cells with the granularity needed to distinguish them.

Finally, we provided a resource to better understand the V-SVZ neurogenic niche and its markers, thereby aiding the enrichment of a new qNSC population and supporting future research aimed at manipulating them. Such a resource could be crucial for activating NSCs following neuronal damage, offering clinical potential to treat neurodegenerative disorders.

## Supplementary information

Supplementary Table S1

Supplementary information

## Data Availability

The paper and Supplementary information contain all the data needed to evaluate this study’s conclusions. The corresponding author provides further details upon reasonable request. scRNA-seq data are deposited in GEO under the accession GSE311603. Figure 1 b, e data were extracted from Dulken et al.^8^. This data pertains to a scRNA-seq atlas of the murine V-SVZ. No new codes were generated for the analysis presented here.
