# Supplementary material for: Identification of a novel deeply quiescent neural stem cell population in the subventricular zone: a potential source for brain repair
Source: Cell Discov. 2026 Jul 14;12:52. doi: 10.1038/s41421-026-00914-4 (PMC13365591; doi:10.1038/s41421-026-00914-4)
Supplement: Supplementary file 2 — Supplementary information [file 41421_2026_914_MOESM2_ESM.pdf]

Supplementary Information

Identification of a novel deeply quiescent neural stem cell population in the subventricular zone: a potential source for brain repair.

Arup R. Nath, Sanskar Ranglani, Mohd Yaseen Malik, Jacek Szymanski, Anis Uz Zaman, Emmanouela Repapi, Roy Drissen, Natalie M. Doig, Peter J. Magill, Claus Nerlov, and Liliana Minichiello*

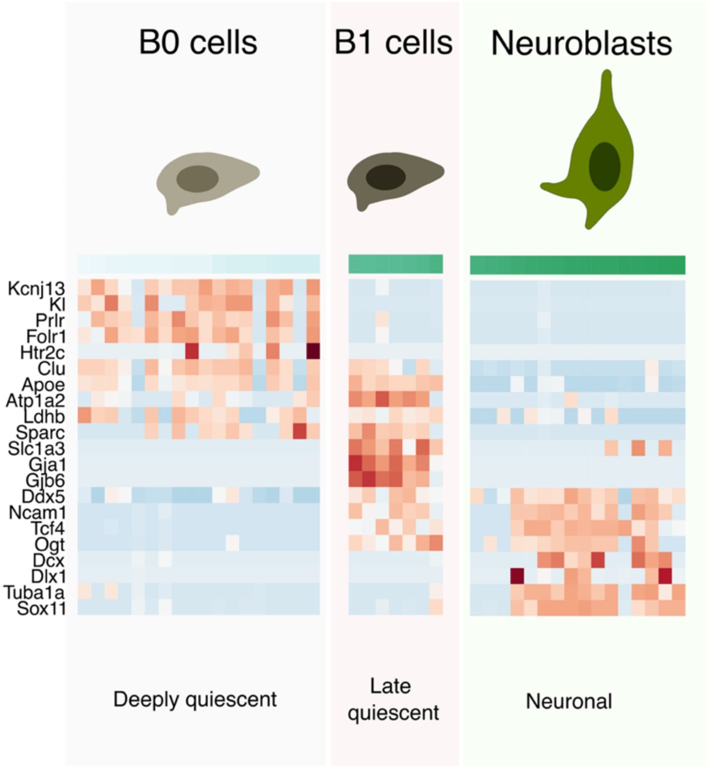

Graphical abstract: This study discovers a previously unappreciated population of deeply quiescent neural stem cells in the brain's subventricular zone. These cells exhibit unique genetic characteristics distinct from those of known stem cells. They express *Htr2c* and *Prlr* and are identified by *Kcnj13*, which encodes the inward-rectifying potassium channel KiR7.1. This discovery opens new avenues for research into brain repair and neurodegenerative diseases.

Supplementary Text

Characterisation of the *Penk-Cre^{Ai9}* line using canonical V-SVZ neurogenic niche markers

Canonical ventricular-subventricular (V-SVZ) neurogenic niche markers were used to highlight qNSCs, neural progenitors (NPCs), and neuroblasts (NBs) and to characterise the identity of the enkephalinergic-derived tdTomato^{+} cells in the V-SVZ. Surprisingly, we found that most tdTomato^{+} cells in the V-SVZ from the *Penk-Cre^{Ai9}* line colocalised with markers for all these cell types, namely the qNSC marker GFAP[1] with a typical bipolar morphology (Fig. S1d-f), the NPC marker Ki67[2] (Fig. S1g-i), and the NB marker SOX11[3] (Fig. S1j-l). Whereas colocalisation with an ependymal cell marker, CD133, which has been inconsistently shown to label some qNSCs of the V-SVZ[4], was found to only minimally overlap with tdTomato^{+} cells (Supplementary Fig. S1m-o). Overall, of the total tdTomato^{+} cells found in the V-SVZ of the *Penk-Cre^{Ai9}* line (about 15000 cells lining the lateral wall) (Fig. S1p), the highest proportion (48%) colocalised with GFAP, suggesting that about half of these cells express a quiescent marker of NSCs, followed by SOX11 (27%), and Ki67 (20%). The latter two markers confirmed that a quarter of tdTomato^{+} cells represent a subset of terminally differentiating neuroblasts in the ventricular-V-SVZ. Finally, the CD133 marker showed negligible overlap (0.7%) with tdTomato^{+} cells, indicating that the latter were not ependymal (Fig. S1p).

Furthermore, we previously demonstrated that our *BAC-Penk-Cre* line crossed with a reporter line does not exhibit recombination in the striato-pallidal neural epithelium (primarily the lateral ganglionic eminence, LGE) at E11, consistent with ENK expression in differentiating multipotent progenitors but not in earlier progenitors[5]. Therefore, to associate these V-SVZ tdTomato^{+} cells' embryonic origin with qNSCs, we performed a time-course analysis to capture ENK's initial expression in the LGE of the *Penk-Cre^{Ai9}* reporter line. Consistent with reports that NSCs are identified as early as E11.5[6], we found reporter expression in the *Penk-Cre^{Ai9}* line at E11.5 and thereafter in the LGE (Fig. S2a-f).

Moreover, as the NSCs from the V-SVZ migrate along the rostral migratory stream (RMS) to give rise to different classes of interneurons in the olfactory bulb (OB)[7], we then reasoned that such interneurons must be positive for reporter expression in the *Penk-Cre^{Ai9}* line (Fig. S3a-f). Consistent with our hypothesis, we found that tdTomato^{+} cells colocalised with several major

interneuron subtypes in the OB[8], including parvalbumin (PV), calretinin (CALR), and calbindin (CALB) (Fig. S3g-l). We also observed that tdTomato colocalised with doublecortin (DCX), a marker of migrating neuroblasts, both in the OB (Fig. S3m-n) and the rostral migratory stream (Fig. S3b-f). These findings confirmed that the *Penk-Cre*^{Ai9} line reliably labelled some qNSCs and cells further along the differentiation trajectory of the V-SVZ neurogenic niche.

scRNA-seq of tdTomato^{+} cells from the V-SVZ of the *Penk-Cre*^{Ai9} line uncovers diverse B cell populations

Sorting strategy (see Fig. S13 and methods for details): although we used tdTomato to identify and capture these cells, the mechanical isolation process can damage cells and trigger microglia's engulfment of material from labelled or unlabelled cells[9,10], resulting in a confounding microglial signature. Therefore, we refined our sorting strategy by using the cell-surface antigen CD11b, a widely used and effective marker of microglia in brain tissues[11,12], and collected the tdTomato^{+}/CD11b^{-} population. This allowed us to isolate neurogenic cells that passed quality control and filtering (Fig. S4b).

Genes exclusively expressed in B0 cells included Klotho (*Kl*), which promotes neural stem cell survival, proliferation, and differentiation[13]. Similarly, transthyretin (*Ttr*) was also highly expressed in B0 cells. Its deficit has been shown to induce apoptosis in NSCs in the V-SVZ niche[14]. Sclerostin Domain-Containing Protein 1 (*Sostdc1*), which acts as an antagonist of bone morphogenetic proteins (BMPs) that are known to influence qNSC maintenance and differentiation through extracellular matrix formation[15]. Also, Retinol Dehydrogenase 5 (*Rdh5*), which is involved in the retinoid cycle, participates in the autocrine regulation of NSCs[16] (Fig.S4c).

To determine whether we could consistently isolate these neurogenic cells, we also used an alternative sorting strategy to collect tdTomato^{+} cells, omitting the CD11b antibody, as shown (Fig. S5a-b). Using this dataset (hereafter called "dataset B"), we confirmed consistency across isolated clusters, such as B0 cells and migrating neuroblasts (Fig. S5c-e). Therefore, we leveraged Seurat to integrate our two datasets using the top 2000 genes[17]. Upon such integration and without performing any batch correction, we found that B0 cells from both

experiments clustered together, as did migrating neuroblasts (Fig. S5f). B1 cells (GLAST$^+$ NSCs), which we isolated only in dataset A, clustered near migrating NBs (Fig. S5f).

Immunohistochemical validation of scRNA-seq results

Using well-validated antibodies[18], we confirmed that KiR7.1 expression is restricted to the V-SVZ, as it was nearly undetectable in the striatal parenchyma, suggesting that *Kcnj13* marks only qNSCs of the V-SVZ and not striatal iSPNs (Fig. S6d-f). We then prepared whole-mount sections of the V-SVZ as in previous studies[19] to visualise KiR7.1 expression from a different plane of view. This again showed that KiR7.1 was widely expressed on the walls of the lateral ventricles and that tdTomato$^+$ cells in the V-SVZ displayed a stellate morphology (Fig. S6g-l). Interestingly, but perhaps unsurprisingly, such a stellate shape is characteristic of qNSCs of the V-SVZ. Co-immunostaining for both GFAP and KiR7.1 revealed that some tdTomato$^+$ cells of the V-SVZ were positive for both markers (KiR7.1$^+$/GFAP$^+$/tdTomato$^+$) (Fig. S6m-o). In addition, there were also some KiR7.1$^+$/GFAP$^-$, some KiR7.1$^+$/GFAP$^+$, and some KiR7.1$^-$/GFAP$^+$ cells. The presence of these intermediate stages supports the possibility that the transition between B0 and B1 may occur. We also validated the expression of the prolactin receptor (PRLR), another marker of B0 cells predicted by scRNA-seq, by immunostaining, which yielded similar results: immunostaining was restricted to the V-SVZ and colocalised with tdTomato$^+$ cells in some regions (Fig. S6p-r). Finally, we validated a marker of B1 cells, the canonical marker of qNSCs, GLAST (Fig. S6s-u)[4], which also colocalised with some tdTomato$^+$ cells.

B0 cells are quiescent and do not decrease in response to treatment with an anti-mitotic agent

Since tdTomato in the *Penk-Cre^{Ai9}* line labels a range of cell types across different developmental stages within the V-SVZ, including astrocytic-like (qNSCs), we aimed to functionally validate these findings. Because the use of primary NSC cultures as neurospheres to demonstrate their potential has been debated and has been shown to exhibit essential differences between *in vitro*-cultured neurospheres and *in vivo* NSCs, including differences in inflammatory expression markers[20], we chose two distinct *in vivo* functional experiments. First,

we treated a group of mice with 5-fluorouracil (5-FU), an antimitotic drug that selectively reduces proliferating cells while sparing non-proliferating cells[21]. We reasoned that, if any tdTomato$^+$ cells on the walls of the V-SVZ survive such treatment, they would be qNSCs, in contrast to those further down the differentiation continuum, such as NPCs. This experiment confirmed that, while 5-FU treatment significantly reduced the total number of proliferating Ki67$^+$cells and tdTomato$^+$/Ki67$^+$ cells in the V-SVZ, it did not significantly affect the number of tdTomato$^+$/GFAP$^+$ or tdTomato$^+$/KiR7.1$^+$ cells in the V-SVZ (Fig. S7a-k). Importantly, the expression of KiR7.1 quantified through immunostaining intensity was unchanged between treated and untreated animals (Fig. S7e, f, k), corroborating the existence of B0 cells (KiR7.1$^+$/tdTomato$^+$ and KiR7.1$^+$/tdTomato$^-$) qNSCs state in addition to the canonical B1 cells (GFAP$^+$).

Penk-Cre^{Ai9} **V-SVZ tdTomato$^+$ cells comprise qNSCs that can be activated following neuronal injury**

We then asked whether tdTomato$^+$ cells in the V-SVZ of the *Penk-Cre^{Ai9}* line could be activated in response to neuronal injury, as previously reported in other studies [22,23,24]. Therefore, we created a partial progressive model of Parkinson's disease by performing ipsilateral stereotaxic injections of 6-OHDA into the striatum of the *Penk-Cre^{Ai9}* line to generate a neuronal lesion[25]. We labelled their brains with a proliferation marker BrdU[26] (Fig. S8a). Tyrosine hydroxylase (TH) immunostaining confirmed successful ablation of dopaminergic innervation to the striatum on the ipsilateral side of the injection (Fig. S8b). Finally, BrdU staining revealed that on the ipsilateral side of the injection, there was a selective and unequivocal increase in BrdU$^+$ proliferative cells, also including a significant rise in BrdU$^+$/tdTomato$^+$ cells (Fig. S8c-g). This increase could not be attributed to an *in situ* increase in *Penk* expression, as RNA-seq of striata from a 6-OHDA-induced partial progressive model of Parkinsonism has shown that *Penk* expression decreases following 6-OHDA induction compared with vehicle-treated[27]. Therefore, it would be difficult to explain the presence of BrdU$^+$/tdTomato$^+$ cells in the striatum of 6-OHDA-treated *Penk-Cre^{Ai9}* mice unless tdTomato$^+$ cells from the SVZ are activated in response to neuronal damage and migrate into the striatum. These experiments confirmed that some tdTomato$^+$ cells in the *Penk-Cre^{Ai9}* line are quiescent and can be activated in response to neuronal injury, thereby supporting striatal tissue repair.

Moreover, neuroblasts in the V-SVZ migrate along blood vessels, which guide their movement and release signals that aid their migration[23]. Although under normal physiological conditions, neuroblasts rarely leave the RMS to enter the striatum, we reasoned that tdTomato^{+} cells should colocalise with blood vessels in the V-SVZ in the *Penk-Cre*^{Ai9} line. Therefore, to highlight blood vessels, we stained for CD31 and found that some tdTomato^{+} cells indeed colocalise with blood vessels, which clearly contact the V-SVZ in the Penk-Cre^{Ai9} line and are thus potentially migrating along the striatal vasculature (Fig. S8h-j). To support these findings and to highlight neuroblasts entering the striatum under physiological conditions, albeit to a lesser extent, we imaged sections from 2M-old Tg(Dcx-EGFP)BJ224Gsat/Mmmh mice, which express EGFP under the control of the DCX promoter, thereby labelling DCX+ neuroblasts (Fig. 8k-p). The images in Fig. S8k-p show clear examples of neuroblasts (highlighted by DCX-EGFP colocalising with DCX antibodies) migrating along the rostral migratory stream, as expected, as well as examples of neuroblasts entering the striatal parenchyma.

Functional validation of B0 cells

Lastly, we sought to determine whether B0 cells could be functionally modulated and how this modulation would affect neurogenesis. Since some B0 cell markers identified in this study were previously associated with qNSC function, for example, *Prlr* and *Htr2c*[28,29], we found several studies in which, in the absence of the present knowledge, some B0_cell markers have been manipulated *in vivo* and *in vitro*, demonstrating diverse effects on the proliferation and migration of NSCs and neuroblasts along the V-SVZ-RMS-OB axis (Table S2). Specifically, Tong *et al.* (2014)[28] have shown that treatment with a 5-HT2C receptor agonist (encoded by *Htr2c*) or antagonist, respectively, increases or decreases the number of BrdU-labelled proliferating cells in the murine V-SVZ. Similarly, Wang *et al.* (2013) reported that subcutaneous administration of prolactin, which activates PRLR (encoded by *Prlr*), increases both BrdU^{+} proliferating cells in the V-SVZ and NeuN^{+}/BrdU^{+} newborn neurons in the OB[30]. Consistently, Shingo *et al.* (2003) reported that both subcutaneous and intracerebroventricular administration of prolactin in wild-type mice induces BrdU^{+} proliferating cells in the SVZ and newborn interneurons in the OB[31]. Importantly, Shingo *et al.* (2003) and Mak *et al.* (2007) demonstrated that both heterozygous and homozygous *Prlr* knockout mice exhibit reduced

numbers of $BrdU^+$ proliferating cells in the SVZ under prolactin-inducing conditions compared with wild-type controls[31,32] (Table S2). In addition, Kim *et al.* (2025) found that *in vitro* knockdown or overexpression of *Kl* (encoding Klotho) results in smaller or larger neurospheres, respectively, indicating altered NSC proliferative capacity[13]. Finally, Vancamp *et al.* (2019) showed that transthyretin knockout ($Ttr^{-/-}$) mice display fewer DCX^+ migrating neuroblasts in the V-SVZ than wild-type animals[33].

Collectively, these findings demonstrate that modulation of B0 cells, either through activation of specific receptor markers (e.g., 5-HT2C, PRLR) or alteration of genes selectively expressed in B0 cells (e.g., *Kl, Ttr*), directly influences proliferation and migration within the V-SVZ neurogenic niche and yields newborn OB interneurons. These results provide functional support for our findings and strongly suggest that B0 cells, in response to a stimulus, can activate neurogenesis via proliferative progenitors ($BrdU^+$), which subsequently differentiate into migrating neuroblasts and ultimately mature OB interneurons ($BrdU^+$/$NeuN^+$/DCX^+). Hence, our study identifies B0 cells as a population distinct from $GLAST^+$ B1 cells and characterised by a more quiescent transcriptional profile.

B0 cell markers expression increases with ageing

It has been reported that with ageing, NSCs in the SVZ become more quiescent[34]. Given that B0 cells appeared more quiescent than canonical B1 cells (Fig. 1, Fig. S4), we hypothesised that B0 cell marker expression levels would increase with age, supporting their deeper state of quiescence. We previously performed bulk RNA sequencing on $tdTomato^+$ cells from *Penk-Cre*^{Ai9} mice at 3M or 8M of age[35]. Reanalysis of this dataset revealed that the expression of B0 cell markers (*Kcnj13*, *Prlr*, *Kl*) increased significantly with age (Fig. S19a-d). However, markers of B1 cells (*Slc1a3*, *Id3*) did not exhibit such age-related upregulation (Fig. S19e-f). These findings suggest that the age-associated increase in NSC quiescence is linked to elevated expression of B0 cell markers rather than to B1 cell markers.

Single-nucleus RNA-seq of the adult human V-SVZ reveals that *KCNJ13*$^+$ NSCs are less differentiated

To identify B0 cells in the human V-SVZ, we reanalysed the single-nucleus RNA-seq dataset generated by Puvogel *et al.* (2024)[36] (Fig. S10a). We visualised the expression of canonical markers of V-SVZ/striatal cell types for each identified cluster and confirmed their identity (Fig. S10b). We then distinguished *KCNJ13*$^+$ from *KCNJ13*$^-$ NSCs and added a metadata label indicating whether *KCNJ13* expression was >0 or <0 (Fig. S10c). Finally, we performed differential gene expression analysis of *KCNJ13*$^+$ NSCs vs *KCNJ13*$^-$ NSCs, followed by pathway analysis. Gene ontology analysis of genes downregulated in *KCNJ13*$^+$ cells compared with *KCNJ13*$^-$ cells revealed pathways including neuronal migration, axonogenesis, neurogenesis, and nervous system development and projection (Fig. S10d). This confirmed our results from the *Penk-Cre*^{Ai9} line, suggesting that *KCNJ13*$^+$ NSCs are less differentiated and thus that this process may be conserved across the mammalian neurogenic niche of the V-SVZ.

Experimental validation of single-cell data by isolating B0 cells using KiR7.1 antibodies

Finally, we asked whether we could use an antibody raised against KiR7.1, encoded by the *Kcnj13* gene, to isolate and validate the existence of a specific NSC state, such as B0 deeply qNSCs, and to enable future studies allowing modulation of these cells. Therefore, we microdissected the lateral walls of the lateral ventricles from 2-month-old *PenkCre*^{Ai9} mice, as shown in Fig. S4a. From dissociated tissues using our established FACS-sorting strategy[37], we collected 10 cells in triplicate from each biological replicate (n = 3) of each sorted population, namely KiR7.1$^+$/tdTomato$^+$/CD11b$^-$ and KiR7.1$^-$/tdTomato$^+$/CD11b$^-$. The former should contain mainly B0 cells, identified by the KiR7.1 antibody and tdTomato, and the latter should comprise all other tdTomato$^+$ cells from the *Penk-Cre*^{Ai9} line, including B1 cells, neuroblasts, iSPNs, and some B0 cells identified by tdTomato alone. We then performed high-throughput microfluidic analysis as previously described[38]. TaqMan Gene Expression Assays were used in quantitative real-time PCR (qPCR) to measure gene expression and assess whether the transcriptional signatures of the sorted populations were consistent with those predicted by the scRNA-seq data. As shown in Fig. S11, KiR7.1$^+$/tdTomato$^+$/CD11b$^-$ cells, compared to the KiR7.1$^-$/tdTomato$^+$/CD11b$^-$ population, expressed high levels of *Kcnj13*, *Prlr*, and *Enpp2* as expected, and were negative for *Penk* and *Sox11*, while minimally expressing *Slc1a3*. Such a transcriptional profile aligned with the prediction made by the scRNA-seq data (Figures 1 and

Supplementary Fig. S4), supporting experimental validation of the single-cell analysis prediction and confirming that antibodies raised against KiR7.1 can be used to isolate B0 cells from the murine V-SVZ.

Embryonic origin of adult B0 neural stem cells: proposed model.

In our immunological experiments, we identified some KiR7.1$^+$/tdTomato$^+$ cells (B0 population) that also colocalise with GFAP (B1 population; shown in Fig. S1 and S6). However, using scRNA-seq and trajectory inference (pseudotime), we observed transient gene expression across cell states (Fig. 1). In particular, the downregulation of deeply quiescent gene markers, such as *Klotho* (*Kl*) and *Kcnj13*, in B1 cells, and the upregulation of *Slc1a3* (GLAST) in the same, for example, represent a temporary gene expression state a cell passes through when transitioning between two stable cell states, indicating a transition from B0 to B1 cells.

But we also found KiR7.1$^+$/tdTomato$^+$ cells that do not colocalise with GFAP, supporting the idea that the novel B0 cells, derived from the enkephalinergic lineage and encompassing all steps of the differentiation continuum, constitute an independent lineage from the canonical B1 cell precursors derived from a subset of neural progenitor cells/radial glia that are specified and set aside during embryonic development (E13.5 and 15.5)/neonatal period remaining largely quiescent until they become reactivated postnatally, as originally shown by Merkle *et al.* (2004)[39] and Fuentealba *et al.* (2015)[6]. In other words, B1 populations could also derive from other independent lineages, including the enkephalinergic lineage through B0. Hence, we identified KiR7.1$^+$/tdTomato$^+$/GFAP$^+$ and KiR7.1$^-$/tdTomato$^-$/GFAP$^+$ cells.

To investigate the ontogenesis of B0 cells and shed light on the developmental origin of the adult B0 cells in the V-SVZ, we then performed experiments using a B0 cell-specific marker, such as *Kcnj13*. Using KiR7.1 immunofluorescence staining of the lateral ganglionic eminence (striatal neuroepithelium) at embryonic day 12.5 (E12.5) in sections from the *Penk-Cre*^{Ai9} line showing tdTomato$^+$ fluorescence, we identified different types of progenitors: one in which both tdTomato and KiR7.1 colocalised, and two other types in which tdTomato and KiR7.1 expression were exclusive. These data suggested that KiR7.1$^+$ identifies more than one type of B0 cell, including an enkephalinergic type. Therefore, to corroborate these results, we first performed bioinformatics analysis using a dataset spanning developmental stages from E11.5

to P112, drawn from an integrated single-cell atlas of striatal development by Anderson *et al.* (2023)[40]. As shown in Fig. S12d, the non-proliferating neural progenitor cluster expresses quiescent B0 markers, including *Kcnj13*, *Folr1*, and *Ttr*, while exhibiting minimal expression of the proliferative marker *Mki67*. In contrast, as these cells transition to proliferating progenitors (*Mki67*+), expression of B0-associated markers decreases. *Penk* is minimally expressed in the non-proliferating neural progenitor cluster, consistent with a transitional state in which cells are gradually downregulating *Penk* as they acquire B0 identity. To narrow down the earliest stage at which B0 progenitors would appear, we then analysed an E11.5 dataset from Chen *et al.* 2017[41] and found that *Kcnj13* expression is already present at E11.5 and increases in non-proliferating neural progenitors, where *Penk* is minimally expressed; *Penk* expression increases in proliferating progenitors, together with *Mki67* expression (Fig. S12e). Our results indicate that *Kcnj13* is a reliable novel marker for adult B0 NSCs.

KiR7.1, an inwardly rectifying potassium channel, is best known for its role in ion homeostasis in epithelial tissues (for example, the retina, kidney, and choroid plexus)[18]; its role in early embryonic development is less well characterised, and there is no direct evidence so far that KiR7.1 directly contributes to stem cell quiescence. However, stem cell quiescence is tightly linked to membrane potential (V_{mem}), ion fluxes (K^+, Ca^{2+}, H^+), and metabolic state. Membrane potential is an instructive regulator of cell-cycle state, with hyperpolarisation generally associated with quiescence and depolarisation with proliferation. As potassium channels are the principal determinants of resting membrane potential, they play a central role in controlling cell-cycle entry by modulating voltage-dependent processes, including calcium signalling, cell volume, and intracellular ionic composition[42]. Thus, in principle, any K^+ channel that stabilises a negative V_{mem} could help keep cells in a non-proliferative state. A study by Bjorkgren *et al.* (2021) provides direct electrophysiological evidence that KiR7.1 activity can hyperpolarise cells *in vitro* and *in vivo*[43], and hyperpolarisation is consistent with quiescent states.

We now present a model in Fig. 1g that integrates our results with these findings. As established, indirect pathway spiny projection neurons (iSPNs) develop during embryogenesis from progenitors in the lateral ganglionic eminence (LGE) of the ventral telencephalon. Radial glia cells and intermediate progenitors produce postmitotic neurons that migrate into the

developing striatum. The proposed model suggests multiple origins for adult V-SVZ neural stem cells, in which, between embryonic stages 11.5/12.5, some progenitors, whether enkephalinergic or not, may acquire quiescence through increased expression of the inwardly rectifying potassium channel KiR7.1 and maintain it until later activation.

Supplementary Figures

Figure S1

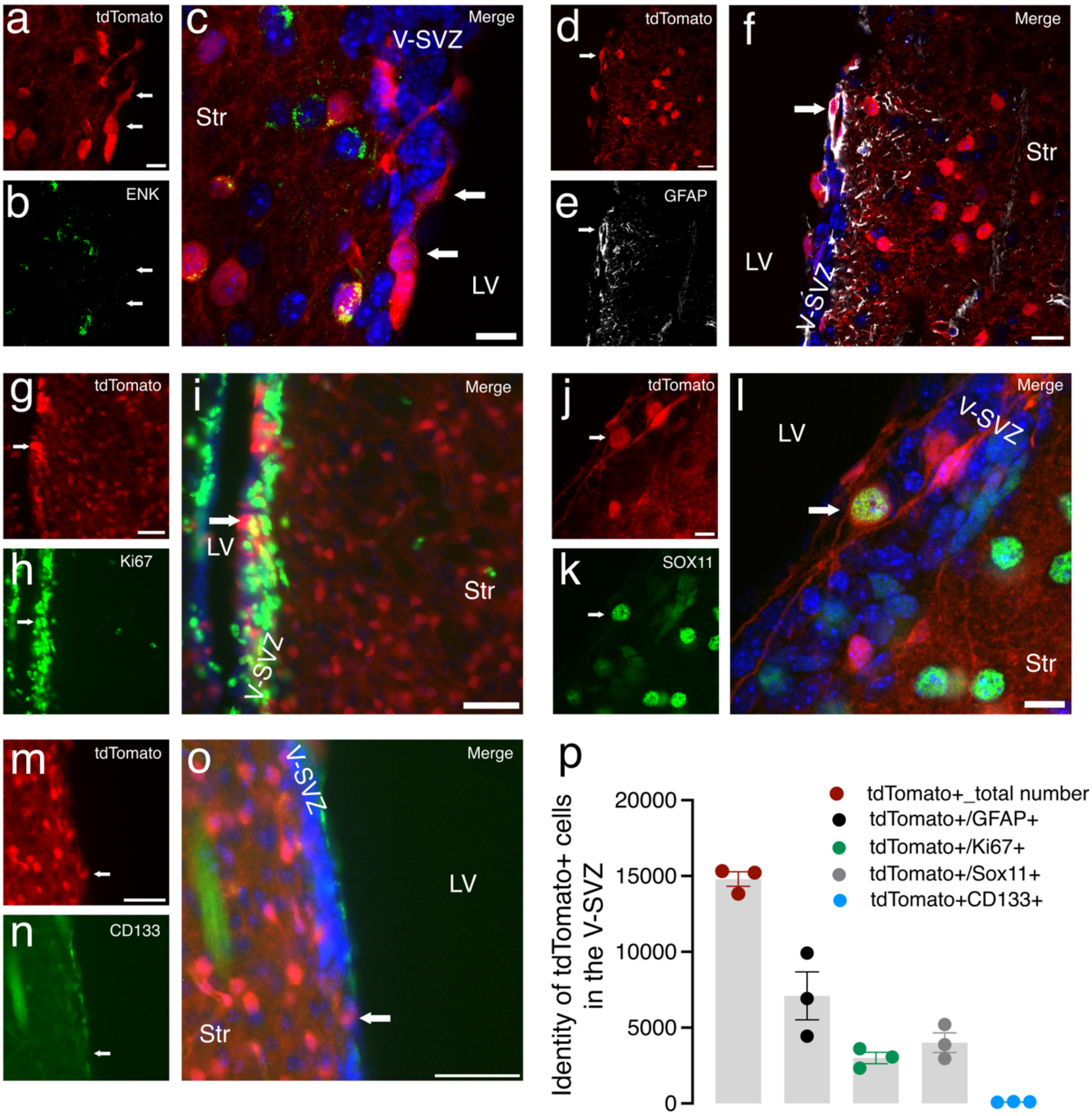

Figure S1, legend: V-SVZ tdTomato^{+} cells from the *Penk-Cre^{Ai9}* line are neurogenic. a, d, g, j, m) Representative tdTomato fluorescence in the V-SVZ and striatal parenchyma. b-c) iSPN marker, ENK (b), and merge with tdTomato and DAPI in blue (c). e-f) Immunostainings for the quiescent neural stem cell marker GFAP (e), and merge with tdTomato and DAPI in blue (f). h-i) Immunostainings for the neural progenitor cell marker Ki67 (h), and merge with tdTomato and DAPI in blue (i). k-l) Immunostainings for the neuroblast marker SOX11 (k), and merge with tdTomato and DAPI in blue (l). n-o) Immunostainings for the ependymal cell marker CD133 (n), and merge with tdTomato and DAPI in blue (o). p) Total tdTomato^{+} cell number

across the V-SVZ of *Penk-Cre*^{Ai9} line (14798±480), number of tdTomato^{+}/GFAP^{+} (7095±1582), tdTomato^{+}/Ki67^{+} (2999±371), tdTomato^{+}/SOX11^{+} (4013±652) and tdTomato^{+}/CD133^{+} cells (105±12). Mice were 14 weeks old, N = 3; data are presented as mean ± S.E.M. LV: Lateral ventricle, V-SVZ: Ventricular-Subventricular zone, Str: Striatum. Scale bars: a-c, 10 μm; d-f, 20 μm; g-i, 50 μm; j-l, 10 μm; m-o, 50 μm.

Figure S2

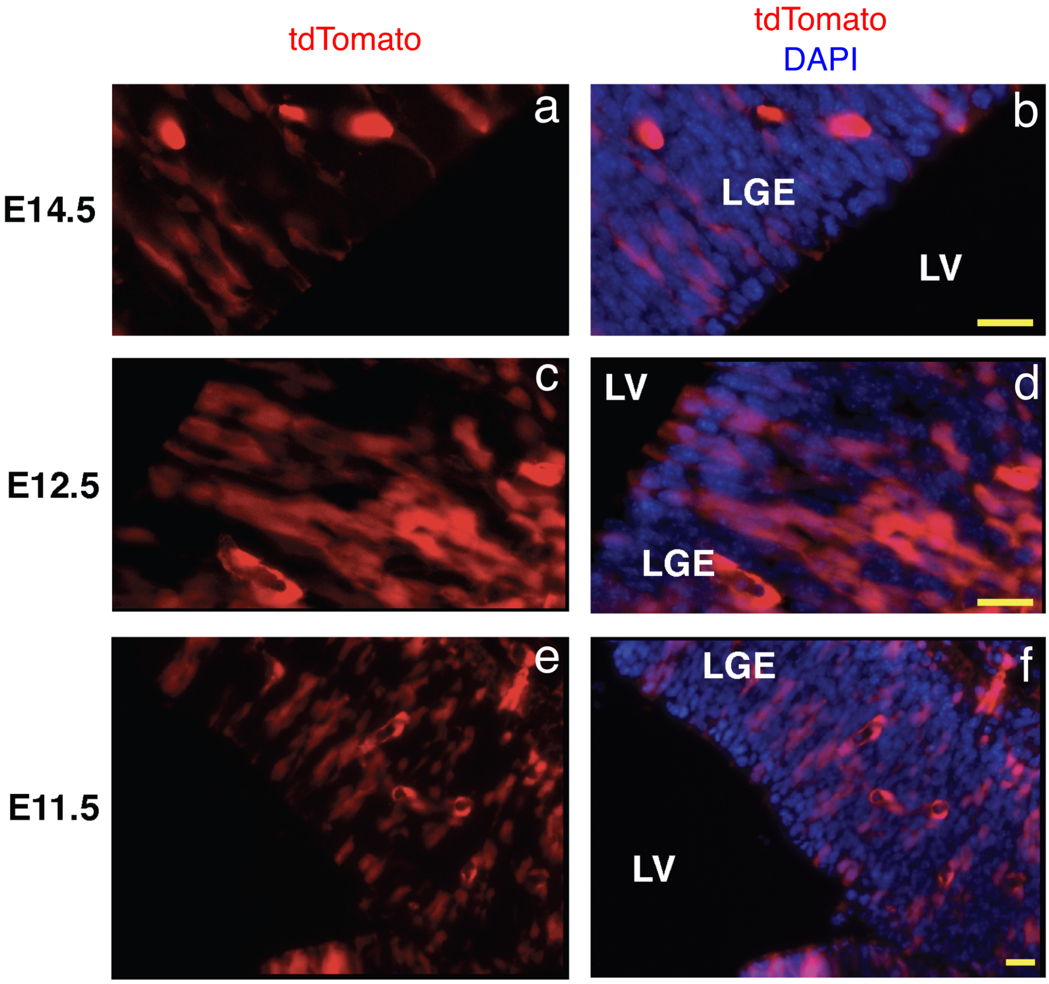

Figure S2, legend: Embryonic origin of tdTomato⁺ cells in the *Penk-Cre*^{Ai9} line.

a-b) tdTomato (a), or tdTomato merged with DAPI in the lateral ventricle (LV) and the lateral ganglionic eminence (LGE) from E14.5 brain of the *Penk-Cre*^{Ai9} line. c-d) tdTomato (c), or tdTomato merged with DAPI in the LV and the LGE from the E12.5 brain of the *Penk-Cre*^{Ai9} line. e-f) tdTomato (e), or tdTomato merged with DAPI in the LV and the LGE from the E11.5 brain of the *Penk-Cre*^{Ai9} line. Scale bars, b, d, f (20 μm).

Figure S3

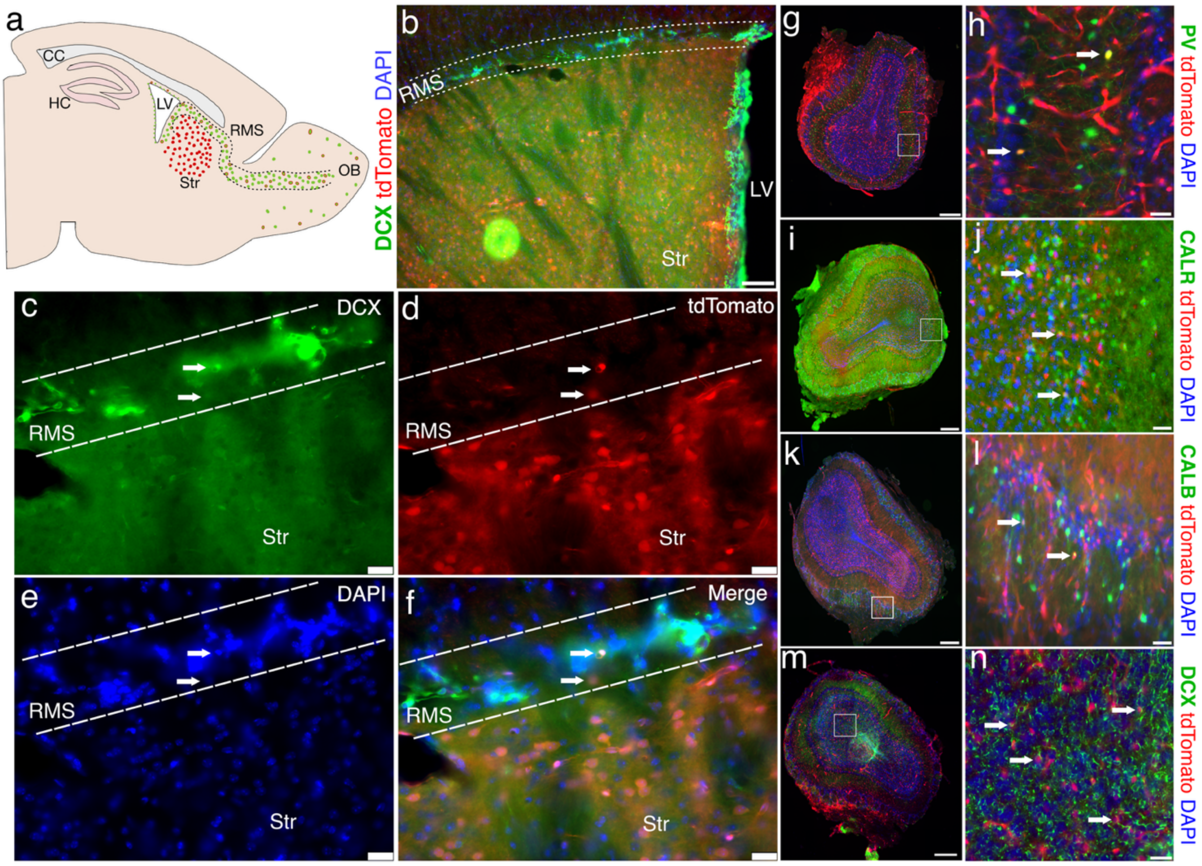

Figure S3, legend: tdTomato⁺ cells from the *Penk-Cre*^{Ai9} line give rise to olfactory bulb interneurons. a) Schematic diagram representing a mouse brain sagittal section, highlighting the DCX+ cells (green) and a subset of tdTomato^{+}/DCX^{+} cells (green fill, red outline cells) in the subventricular zone on the wall of the lateral ventricle (LV) along the rostral migratory stream (RMS) and the olfactory bulb (OB). tdTomato^{+} cells in the striatum (Str)-indirect pathway spiny projection neurons, are also shown. b-f) Representative immunostaining of an adult mouse brain sagittal section stained for DCX, colocalised with tdTomato fluorescence and DAPI (blue). g, i, k, m) Coronal sections of OB immunostained for different interneuron markers. g-h) Representative immunostaining for parvalbumin (PV), merged with tdTomato and DAPI (blue), inset (h). i-j) Representative immunostaining for calretinin (CALR), merged with tdTomato and DAPI (blue); inset (j). k-l) Representative immunostaining for calbindin (CALB) merged with tdTomato and DAPI (blue); inset (l). m-n) Representative immunostaining for doublecortin (DCX) merged with tdTomato and DAPI (blue); inset (n). Mice were 14 weeks old. Scale bars, b, 50 μm; c-f, 20 μm; g, i, k, m, 300 μm; h, j, l, n, 30 μm.

Figure S4

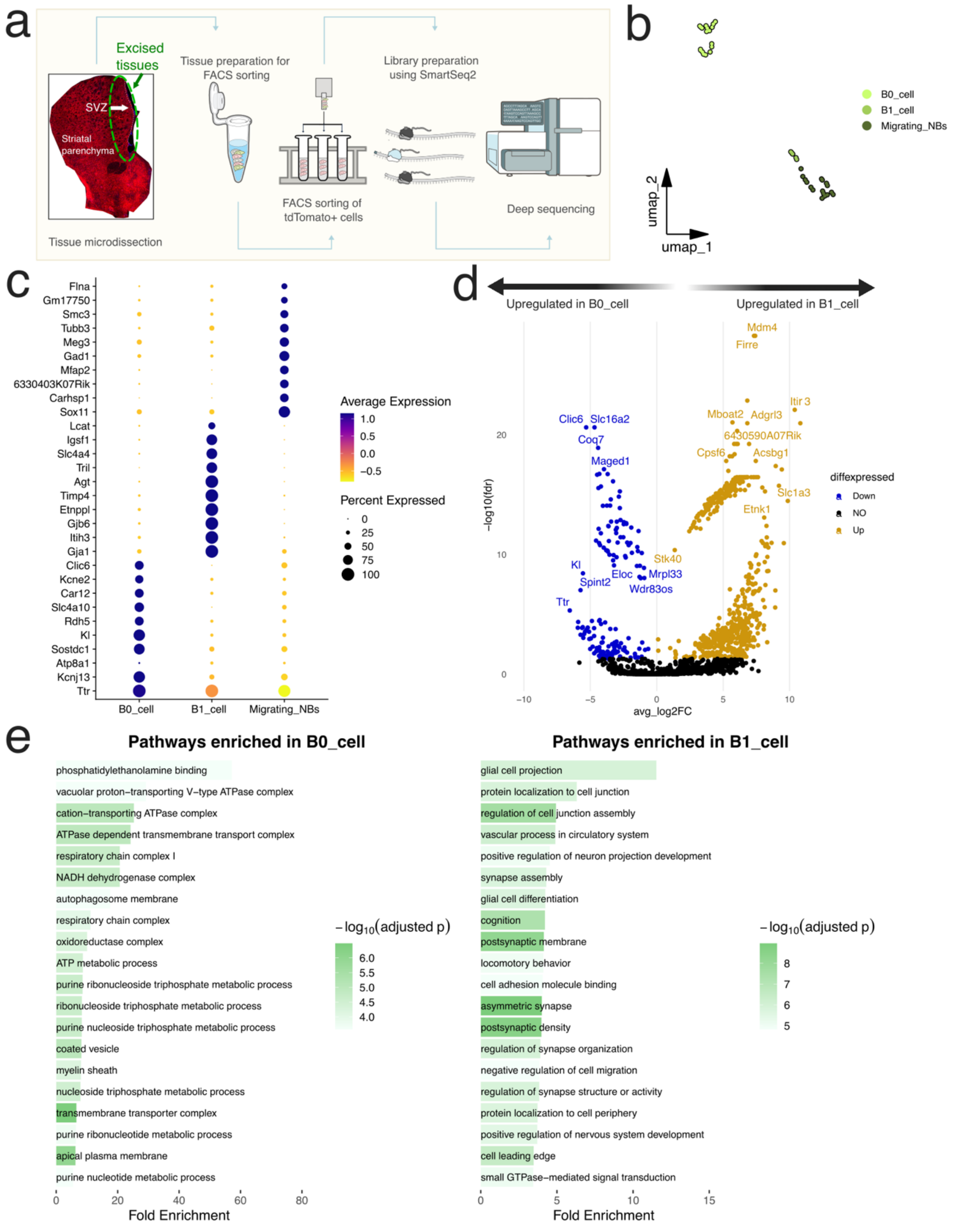

Figure S4, legend: Single-cell RNA-seq of the tdTomato^{+} cells from the *Penk-Cre*^{Ai9} line. a) Summary of experimental paradigm. Microdissected tissue from the *Penk-Cre*^{Ai9} line was prepared into a single cell solution, followed by FACS, library prep, and SmartSeq2 (panel a, generated with NIH BioArt). b) UMAP plot of the clusters captured from scRNA-seq, B0_cell (deeply quiescent neural stem cells of the V-SVZ), B1_cell (late quiescent neural stem cells of the V-SVZ); Migrating_NBs. c) Dot plot of scaled gene expression levels of the top 10 markers

for each cluster, based on differential gene expression analysis (FindAllMarkers, MAST). The size of the dot reflects the proportion of cells in the cluster in which the gene was detected (>0 count), and the dot's colour indicates the average expression z-score. d) Volcano plot demonstrating differential expression output to identify the genes upregulated in B1 cells versus B0 cells with average log2-fold change on the x-axis and - log10 (FDR) on the y-axis. Genes with FDR < 0.05 are coloured either in gold (upregulated in B1 cells) or blue (upregulated in B0 cells). e) Top pathways enriched in B0 cells versus B1 cells (left panel) and B1 cells versus B0 cells (right panel), derived from gene ontology analysis of genes with FDR < 0.05, with the x-axis representing the Fold Enrichment of the pathway and the shading indicating the - log10 of the adjusted p-value. Based on the padj value, all pathways presented here were significantly enriched. UMAP, Uniform Manifold Approximation and Projection.

Figure S5

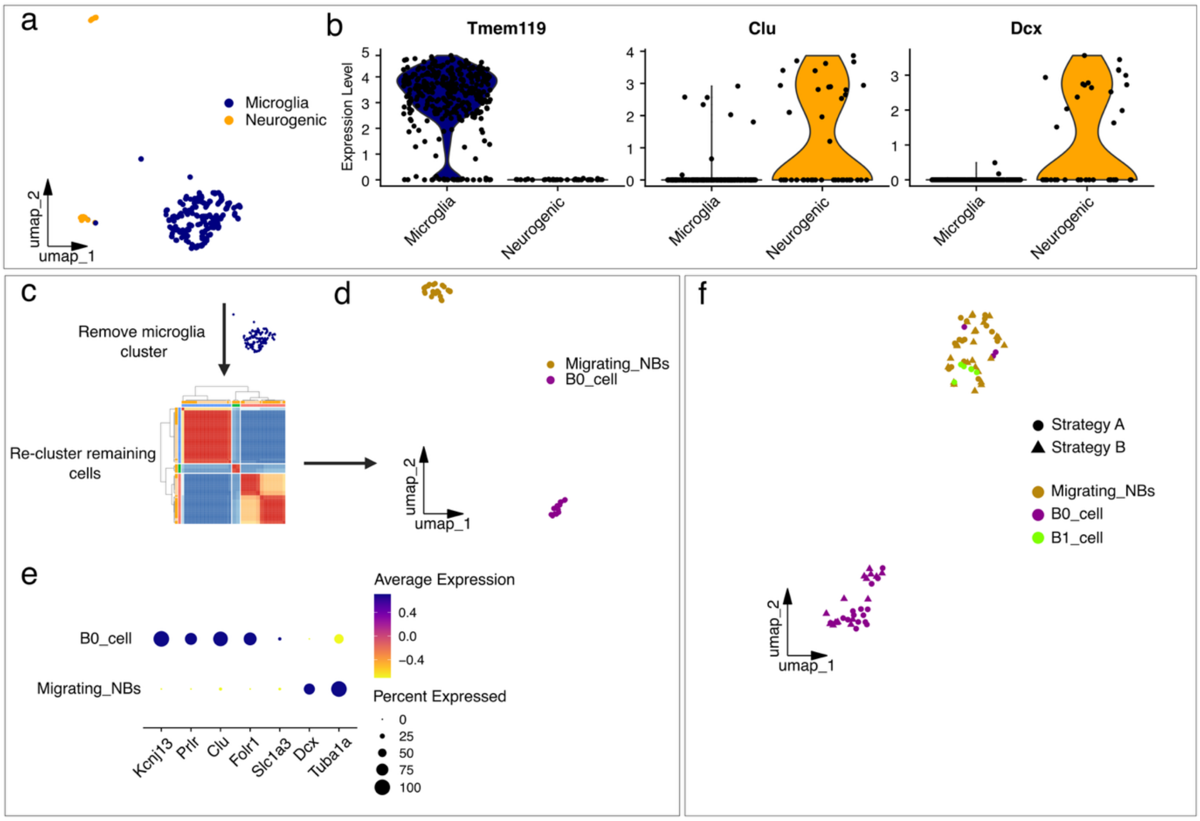

Figure S5, legend: Sorting strategies for scRNAseq of tdTomato^{+} cells from the V-SVZ of the Penk-Cre^{Ai9} line. a) UMAP representation and unsupervised clustering of the single-cell transcriptome from dataset B, where all tdTomato^{+} cells have been sorted, omitting the addition of CD11b, reveals two clusters. Based on differential gene expression analysis, one cluster expressed a microglia signature, while the other exhibited a neurogenic signature. b) Violin plots displaying a canonical marker of microglia (*Tmem119*), which is highly expressed in the microglia cluster. Conversely, markers of neurogenic cells (*Clu* and *Dcx*) were detected in the other cluster and absent in the microglia cluster. c) Removal of the microglia cluster, followed by principal component analysis, dimension reduction, and re-clustering of the remaining cells from dataset B. d) UMAP from re-normalising and unsupervised clustering from dataset B after removal of the microglia cluster, revealing two clusters: deeply quiescent B0 cells and migrating neuroblasts (NBs), similar to those identified in dataset A as shown in panel (e). e) Dot plot displaying scaled gene expression levels of selected markers for the two clusters identified in dataset B. B0 cells from dataset B show a consistent transcriptional profile with B0 cells from dataset A – they express *Kcnj13, Prlr*, *Clu*, *Folr1*, but do not express *Slc1a3* (encoding GLAST). Similarly, migrating NBs cluster expressed markers consistent with migrating NBs in dataset A, *Dcx* and *Tuba1a*. The size of the dot represents the fraction of cells within the cluster in which the gene was detected (>0 count), and the dot's colour represents the average expression z-score. f) A UMAP representation of the integrated datasets indicates that the B0 and migrating neuroblast clusters, identified in both datasets, cluster together.

Colours represent the cell type, whereas the shape indicates the dataset generated by either sorting strategy (see also Fig. S13 and the methods for further details on the sorting strategy).

Figure S6

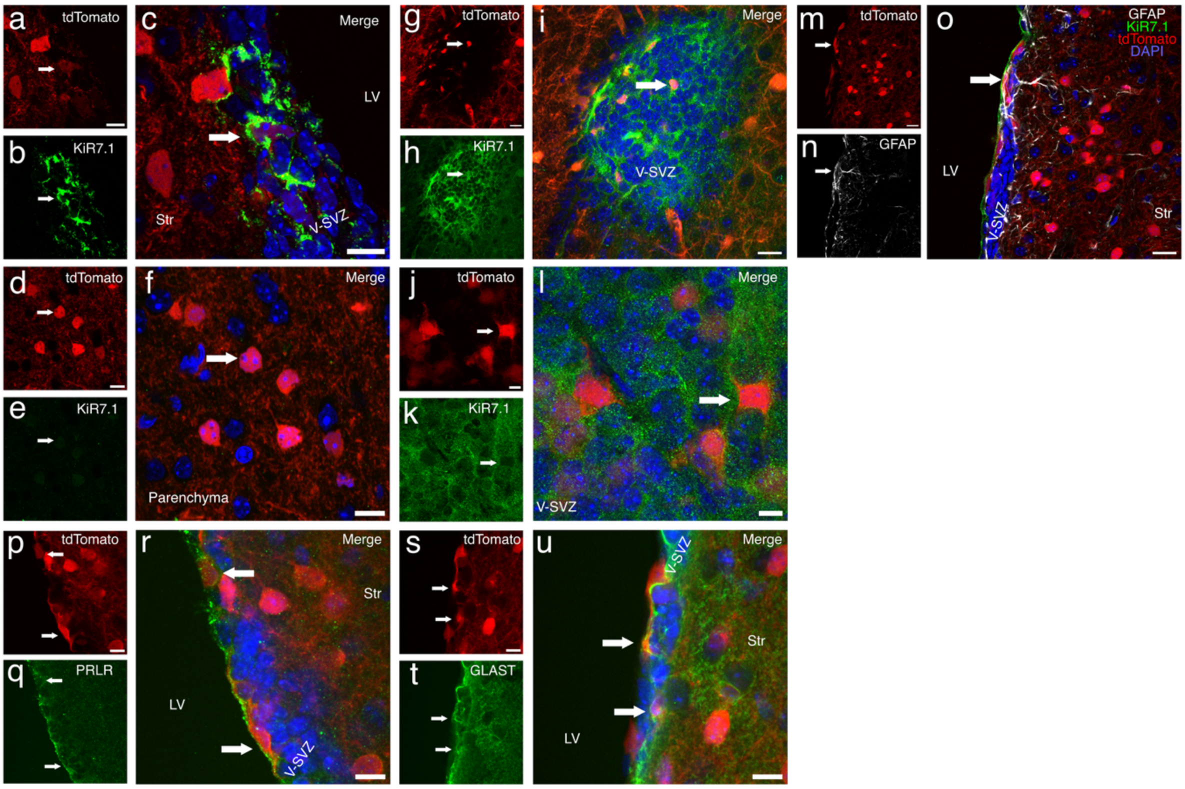

Figure S6, legend: Immunohistochemical validation of heterogeneous B cell markers using the *Penk-Cre^{Ai9} line.* a-c) Representative confocal images for tdTomato on a coronal section visualising the V-SVZ (a), immunostained for B0_cell marker KiR7.1 (b) or merged (c). d-f) Representative confocal images for tdTomato on a coronal section visualising striatal parenchyma (d), immunostained for B0_cell marker KiR7.1 (e) or merged (f), highlighting the absence of KiR7.1 staining in the parenchyma. g-l) Representative confocal images for tdTomato (g,j); B0 cell marker KiR7.1 (h, k); or merge (i, l) on a whole-mount stain of the V-SVZ. m-o) Representative confocal images for tdTomato (m); qNSC marker GFAP (n); or merge with B0_cell marker KiR7.1 (o). p-r) Representative confocal images for tdTomato (p); B0_cell marker PRLR (q); or merge (r) on a coronal section visualising the V-SVZ. s-u) Representative immunofluorescence images for tdTomato (s); B1_cell marker GLAST (t); or merge (u) on a coronal section visualising the V-SVZ. DAPI in blue: c, f, i, l, o, r, u. Scale bars: a, c, d, f, j, l, p, r, s, u, 10 μm; g, i, m, o, 20 μm. V-SVZ: Ventricular-Subventricular zone; LV: Lateral ventricle; Str: Striatum.

Figure S7

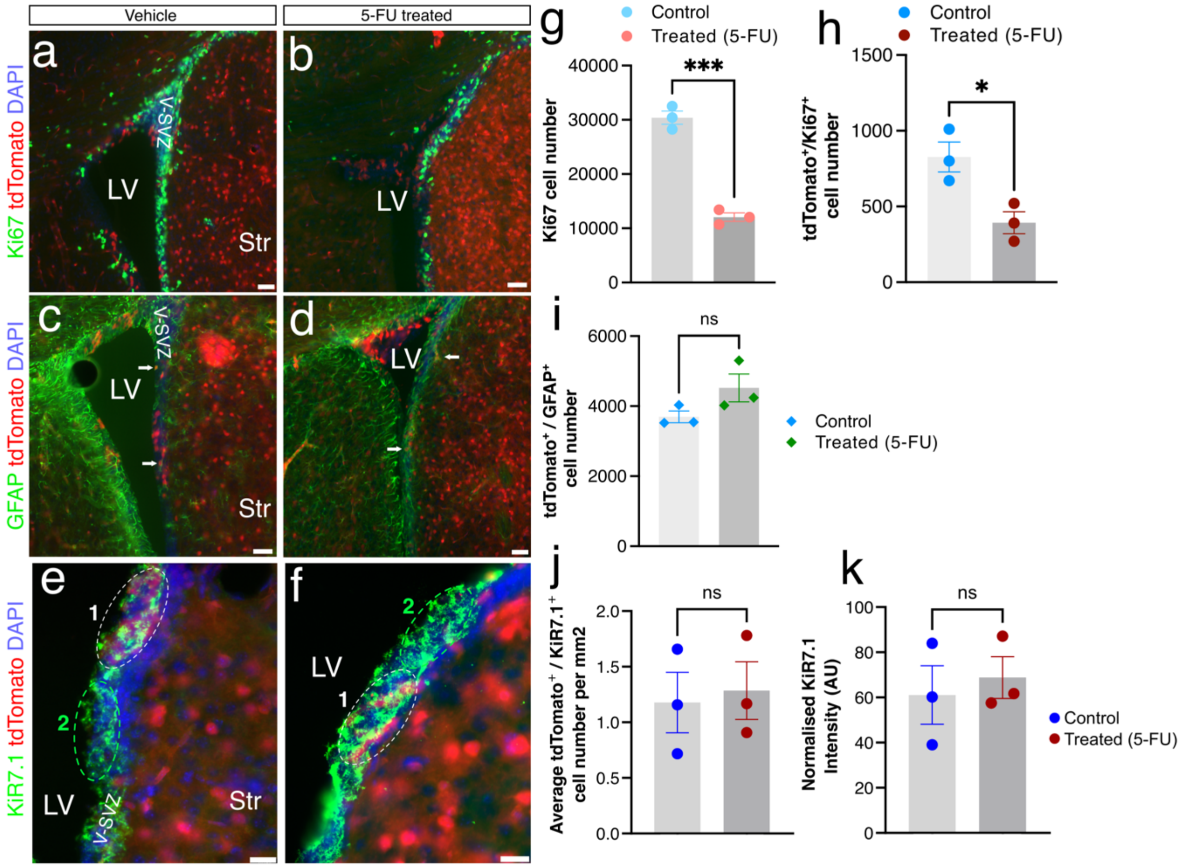

Figure S7, legend: 5-FU treatment does not alter the number of B0 cells in the *Penk-Cre*^{Ai9} V-SVZ. a-b) Representative immunostaining for Ki67, a proliferating cell marker, merged with tdTomato in vehicle-treated or 5-FU-treated mice. c-d) Representative immunostaining for the B1 quiescent neural stem cell marker, GFAP, merged with tdTomato in vehicle-treated or 5-FU-treated mice. e-f) Representative immunostaining of the B0 cell marker, KiR7.1, merged with tdTomato in vehicle-treated or 5-FU-treated mice. Cells within the white circle (1) are patches of KiR7.1+ cells that colocalise with tdTomato, whereas cells highlighted in the green circle (2) are KiR7.1+ cells that are negative for tdTomato. g) Total number of Ki67$^+$ cells in the V-SVZ in vehicle-treated (30397 ± 1230) or 5-FU-treated (12063 ± 788.1) mice, p = 0.0002. h) Number of tdTomato$^+$/Ki67$^+$ colocalised cells in the V-SVZ in vehicle-treated (826.7 ± 99.05) or 5-FU-treated (393.3 ± 72.19) mice, p = 0.024. i) Number of tdTomato$^+$/GFAP$^+$ colocalised cells in the V-SVZ in vehicle-treated (3697 ± 166.8) or 5-FU-treated (4520 ± 395.1) mice, p = 0.13. j) Number of average tdTomato$^+$/KiR7.1$^+$ colocalised cells per mm^2 in the SVZ in vehicle-treated (1.178 ± 0.271) or 5-FU-treated (1.285 ± 0.258) mice, p = 0.7888. k) Normalised KiR7.1 immunostaining intensity (arbitrary units, AU) in the SVZ in vehicle-treated (61.07 ± 12.98) or 5-FU-treated (68.79 ± 9.22) mice, p = 0.656. N = 6 biological replicates per treatment/group and n = 3 per type of immunostaining/genotype; values are means, error bars, S.E.M. *P* values are from an unpaired, two-tailed Student's *t*-test or Welch's test. Scale bars, a-d; 50 µm; e-f; scale bar, 20 µm.

Figure S8

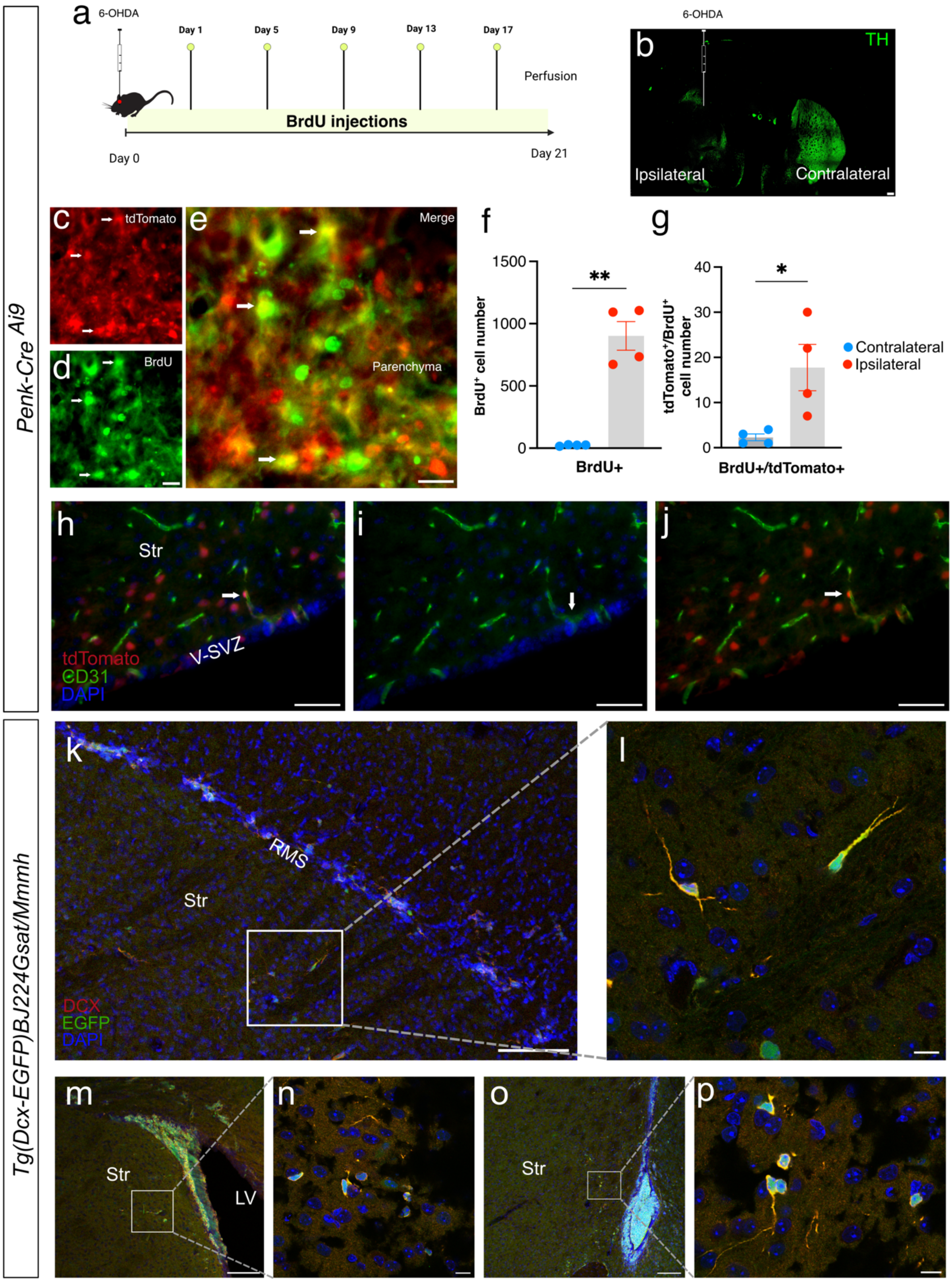

Figure S8, legend: qNSCs in the Penk-Cre^{Ai9} V-SVZ can be activated following neuronal injury and migrate towards the affected area. a) Experimental paradigm (graphic from NIH

BioArt) used to create a partial progressive model of Parkinson's disease. *Penk-Cre*^{Ai9} female mice, 12 weeks old, were injected ipsilaterally with 6-OHDA on day 0, then intraperitoneally injected with BrdU from day 1 every four days, followed by perfusion fixation on day 21. b) A representative coronal section immunostained for TH, showing complete denervation of the striatal afferents on the ipsilateral side while sparing the contralateral side (scale bar: 250 μm). c-e) Representative striatal parenchyma from the ipsilateral side of 6-OHDA injection imaged for tdTomato, BrdU and merge (scale bar: 20 μm). f) Total number of BrdU$^+$ cells in the ipsilateral (901.5 ± 115) or contralateral parenchyma side (23.5 ± 2.6) of the 6-OHDA injection, paired t-test p = 0.0048. g) Total number of BrdU$^+$ cells co-localising with tdTomato$^+$ in the ipsilateral (17.75 ± 5.1) or contralateral parenchyma side (2.25 ± 0.75) of the 6-OHDA injection, p = 0.047. N = 4 biological replicates. Values are means ±S.E.M. *P* values are from paired, two-tailed Student's *t*-test. h-j) Representative images from 2M old *Penk-Cre*^{Ai9} mouse showing tdTomato fluorescence in the V-SVZ and striatal parenchyma. h) tdTomato in red, CD31 in green, labelling blood vessels and DAPI in blue; arrowhead indicates a tdTomato$^+$ cell migrating along a vessel into the striatum. i) CD31 and DAPI; arrow indicates a vessel extending from the SVZ into the striatum. j) tdTomato and CD31; arrowhead indicates a tdTomato$^+$ cell migrating along a vessel into the striatum. Scale bar, h-j: 50 μm. k-l) Representative images from 2M old Tg(Dcx-EGFP)BJ224Gsat/Mmmh mouse, in which EGFP (green) is expressed under the control of the DCX promoter, thereby labelling DCX+ neuroblasts. k-l) Sagittal sections with EGFP in green, DCX immunostaining in red, and DAPI in blue, illustrating neuroblasts migrating from the RMS into the striatum. m-p) Coronal sections showing migration from the dorsal (m) or ventral (o) V-SVZ into the striatum. EGFP signal colocalises with DCX immunostaining, confirming neuroblast identity. Scale bars: k, m, o: 100 μm; l, n, p: 10 μm. RMS: Rostral migratory stream, Str: Striatum.

Table S2: Functional modulation of B0 markers alters V-SVZ/OB neurogenesis *in vivo* and *in vitro*.

Target	Methodology	Results	Reference
***Htr2c* (5HT2C)**	Infusion of 5HT2C agonist for 5 days into the lateral ventricle	Higher number of BRDU+ labelled proliferating cells in the SVZ compared to saline	Tong *et al.*, 2014[28]
	Infusion of 5HT2C antagonist for 5 days into the lateral ventricle	Lower number of BRDU+ labelled proliferating cells in the SVZ compared to saline	
***Prlr* (Prolactin receptor)**	Subcutaneous infusion of prolactin (PRLR stimulant) for 7 days	Higher number of BRDU+ labelled proliferating cells in the SVZ compared to vehicle	Wang *et al.*, 2013[30]
	Subcutaneous infusion of prolactin (PRLR stimulant) for 7 days	Higher number of NeuN+/BRDU+ labelled newborn neurons in the OB compared to vehicle	
***Prlr* (Prolactin receptor)**	Subcutaneous and intracerebroventricular injection of prolactin to WT mice	Higher number of BRDU+ proliferating cells in the SVZ and newborn interneurons in the OB	Shingo *et al.*, 2003[31]
	Prlr $^{-/+}$ mice on gestational day 7 (known to induce prolactin release)	Fewer BRDU+ proliferating cells in the SVZ of Prlr $^{-/+}$ mice compared to Prlr $^{+/+}$ mice	
***Prlr* (Prolactin receptor)**	*Prlr* $^{-/-}$ mice post male-female interaction (known to induce prolactin release)	Fewer BRDU+ proliferating cells in the SVZ of Prlr $^{-/-}$ mice compared to Prlr $^{+/+}$ mice	Mak *et al.*, 2007[32]
***Kl* (KLOTHO)**	siRNA knockdown of *Kl* in NSCs followed by differentiation in-vitro into neurospheres	Reduced the diameter of neurospheres compared to control siRNA	Kim *et al.*, 2025[13]
	Overexpression of *Kl* in NSCs followed by differentiation in-vitro into neurospheres	Increased the diameter of neurospheres compared to control vector	
***Ttr* (Transthyretin)**	*Ttr* knockout mice immunostained for DCX in the SVZ	Lower number of DCX+ cells (neuroblasts) in the SVZ of Ttr KO mice compared to WT mice	Vancamp *et al.*, 2019[33]

Figure S9

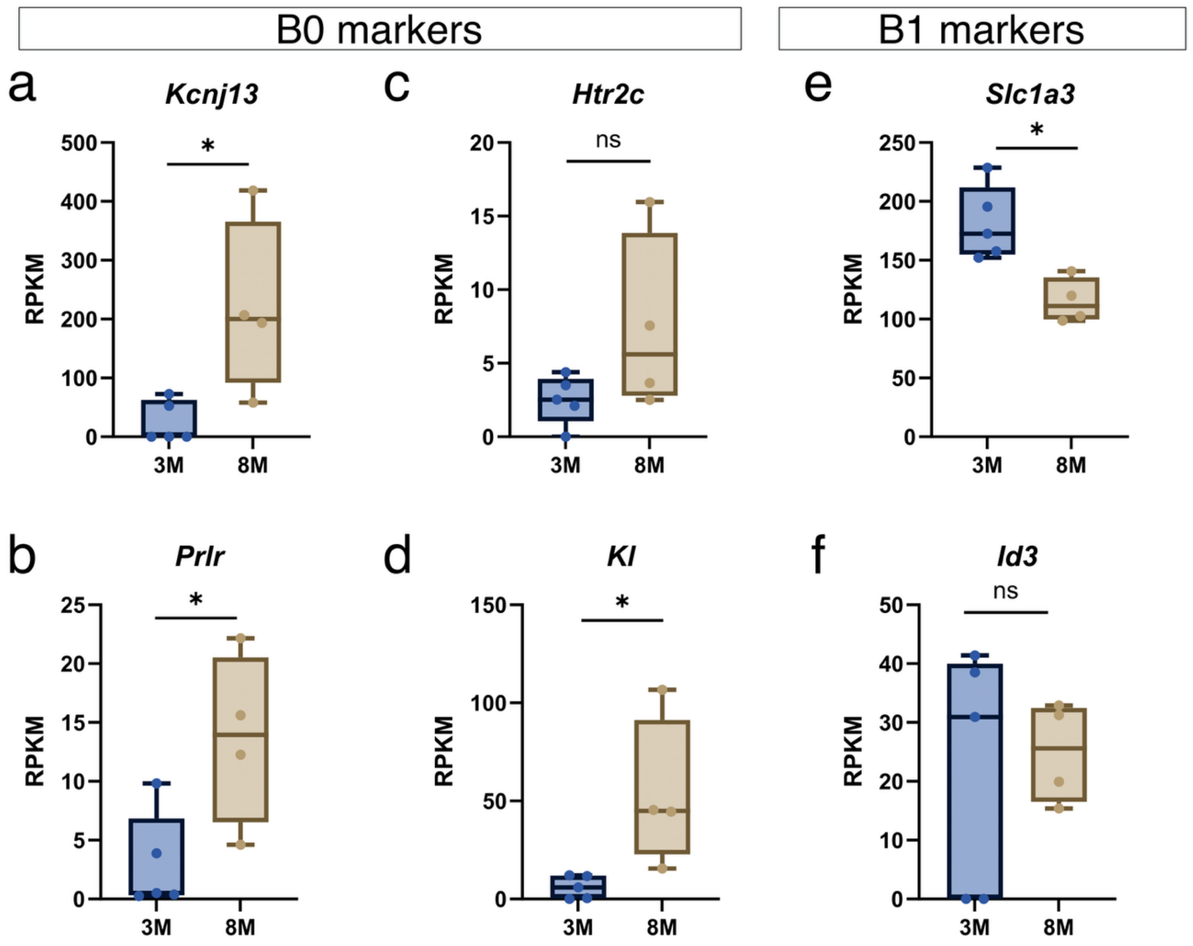

Figure S9, legend: Expression levels of B0 and B1 cell markers during ageing in the *Penk-Cre*^{Ai9} line. Reads Per Kilobase of transcript per Million mapped read values for markers of B0 cells – *Kcnj13* (a), *Prlr* (b), *Htr2c* (c), *Kl* (d); and B1 cells – *Slc1a3* (e), and *Id3* (f). P-value based on Mann-Whitney U-test of ranks. *Kcnj13* p-value: 0.031; *Prlr* p-value: 0.031; *Htr2c* p-value: 0.1905; *Kl* p-value: 0.0159; *Slc1a3* p-value: 0.0159; and *Id3* p-value > 0.999. Data were re-analysed using data from Malik *et al.* (2024)[35]. GEO: GSE221922.

Figure S10

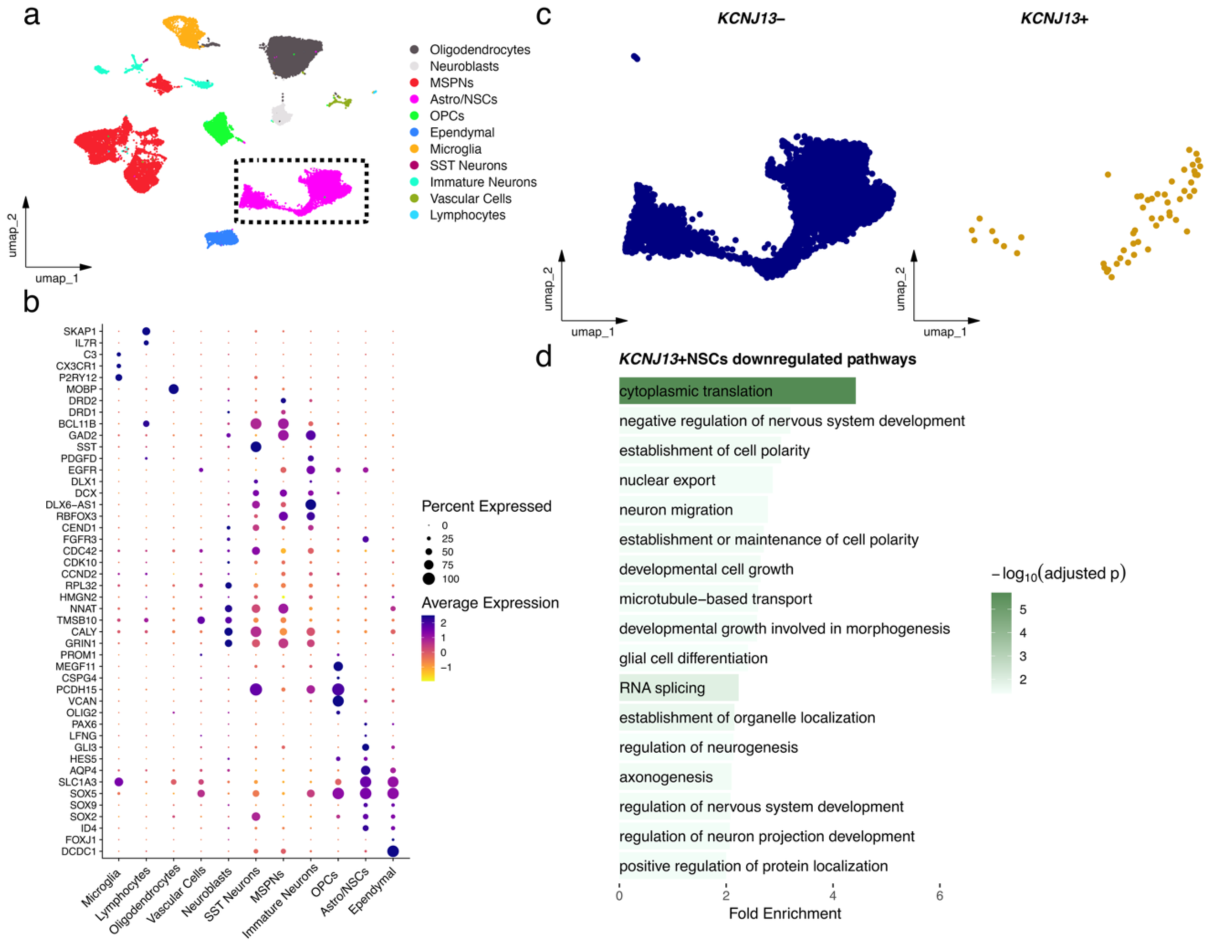

Figure S10, legend: *KCNJ13*⁺ neural stem cells from the human subventricular zone are less differentiated. a) UMAP plot from the Puvogel *et al.*, 2024 dataset reanalysis. b) Canonical markers of each V-SVZ/striatal cell type validating cell type clustering and annotation. c) UMAP plot for Astro/NSCs cluster split by *KCNJ13*$^+$ expression (*KCNJ13* expression > 0). d) Top pathways from gene ontology analysis based on genes downregulated in *KCNJ13*$^+$ NSCs compared to *KCNJ13*- NSCs based on $p < 0.05$. Based on the padj value, all pathways presented here were significantly enriched. The data in this figure were extracted from the GEO dataset (GSE234790); see the method for additional details.

Figure S11

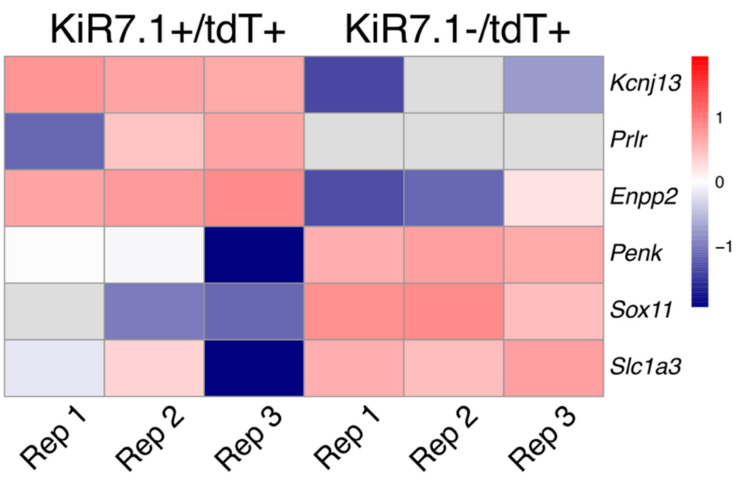

Figure S11, legend: FACS-sorting of KiR7.1^{+}/tdTomato^{+}/CD11b^{-} cells can isolate B0 cells. Scaled gene expression values for *Kcnj13*, *Prlr*, *Enpp2*, *Slc1a3*, *Sox1* and *Penk* across the two populations isolated by FACS. Genes of interest were normalised to *Rn45s* expression. Rep, biological replicate.

Figure S12

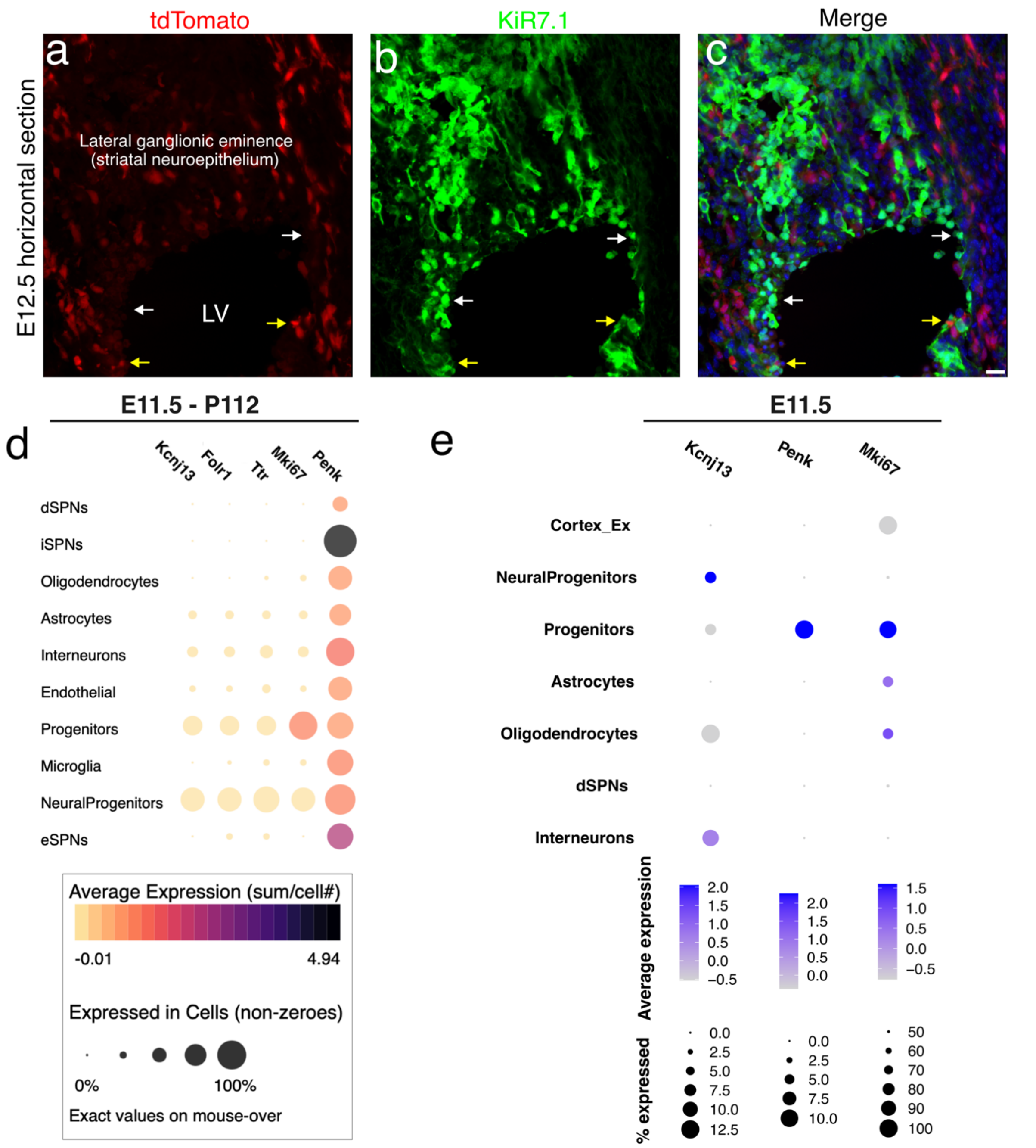

Figure 12 legend: Developmental origin of B0 cells.

a-c) Representative immunofluorescence staining of the lateral ganglionic eminence (striatal neuroepithelium) at embryonic day 12.5 (E12.5), horizontal section from the *Penk-Cre*^{Ai9} line, showing tdTomato (a), KiR7.1 (b), and merged signal with DAPI (c). Scale bar: 20 µm. Yellow arrows indicate examples of KiR7.1^{+} cells co-localising with tdTomato, whereas white arrows indicate examples of KiR7.1^{+} cells lacking tdTomato expression. d) Dot plot of gene expression from an integrated single-cell atlas of striatal development from Anderson *et al* (2023)[40]. Shading indicates the scaled average expression of each gene, while dot size reflects the percentage of cells in each cluster expressing that gene. The plot shows that the neural

progenitor cluster expresses quiescent B0 markers, including *Kcnj13*, *Folr1*, and *Ttr*, while, as these cells transition to proliferating progenitors (*Mki67*+), expression of B0-associated markers decreases. *Penk* expression in the neural progenitor cluster is less abundant than in mature iSPNs. Although the dataset spans developmental stages from E11.5 to P112, the progenitor and neural progenitor clusters are derived predominantly from samples from E11.5 to P9. e) Dot plot of gene expression from Chen *et al.,* 2017[41], highlighting the E11.5 dataset. Shading indicates the scaled average expression of each gene, while dot size reflects the percentage of cells in each cluster expressing that gene. *Kcnj13* expression increases in non-proliferating neural progenitors, whereas *Penk* is minimally expressed and increases in proliferating progenitors together with *Mki67*+ expression.

Figure S13

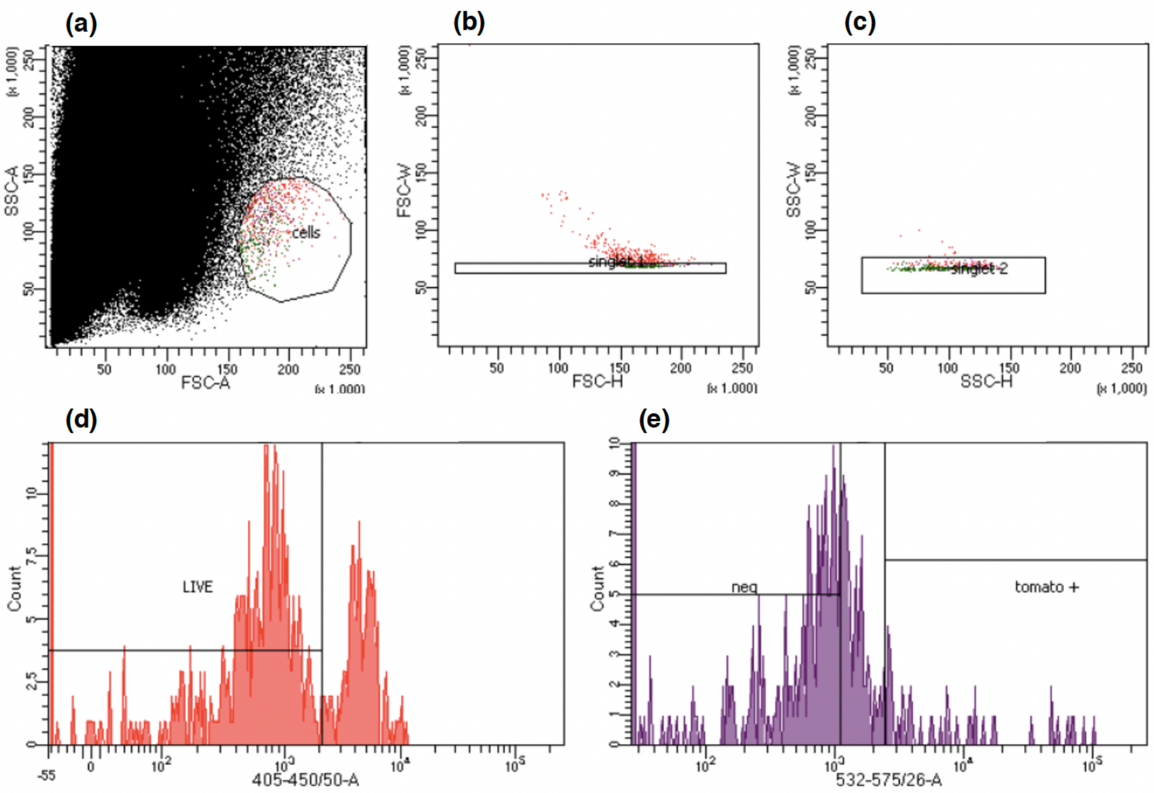

Figure S13 legend. A representative gating protocol for fluorescence-activated cell sorting-mediated isolation and capture of single cells from the *Penk-Cre*^{Ai9} line. (a) Dissociated cells from the *Penk-Cre*^{Ai9} mouse strain as per Fig S4a, stained with LIVE/DEAD stain to identify dead cells. Cells were sorted using a FACSAria Fusion Cell Sorter. All sorts of events are plotted as a function of forward and side scatter. The indicated "cell" gate was chosen following extensive optimisation in previous lab work (Vatanashevannopakorn *et al.* 2017)[37]. Black dots identify all events. Red dots indicate the subset of cells that exhibit the morphological characteristics we identify as cell-specific. **(b)** and **(c)** Forward and side scatter plotted to select strictly for singlets. **(d)** The singlet cells from (b) and (c) are then plotted on a frequency histogram to determine their intensity of LIVE/DEAD staining. Using extensive empirical methods, we determined that live cells are those with fluorescence intensity to the left of the indicated line. **Note:** recovery of live cells after scatter gating of dissociated solid tissues is less efficient, ranging from 20% to 75%, depending on the tissue. CNS tissue is at the more difficult end of the range (due to neuronal connectivity and myelin debris), typically 20-60%. The proportion of live cells in **panel d** is at the very high end of this spectrum. **(e)** The "LIVE" cells from (d) are plotted on a frequency histogram to determine the intensity of the tdTomato fluorescence signal. Cells to the right of the "tomato+" line were taken as Penk-tdTomato^{+} cells. CNS, Central Nervous System.

Materials and Methods

Animals

All animals were kept on a mixed genetic background (C57BL/6J;129). The strains used in this study were the BAC-*Penk-cre*$^{tg/+}$ strain, carrying Cre recombinase under the control of the pre-pro-enkephalin promoter, as previously described[5]. The *Rosa26*-Ai9-*tdTomato*[44] reporter line contains a *loxP*-flanked stop cassette before the *tdTomato* cDNA. The Tg(Dcx-EGFP)BJ224Gsat/Mmmh, transgene insertion BJ224, GENSAT Project at Rockefeller University. Stock Number: 000244-MU. For lineage tracing, the BAC-*Penk*-Cre line was crossed with the Ai9 reporter line, generating control mice carrying both alleles (BAC-*Penk*-*Cre* $^{tg/+}$; Ai9, hereafter referred to as *PenkCre*^{Ai9}). The mice used in this study ranged in age from 5 to 14 weeks. Some embryonic stages used were E11.5, E12.5, and E14.5. Mice had unrestricted access to food and water and were housed in a room with a 12-hour light/12-hour dark cycle (07:00–19:00 h), an ambient temperature of 19–23 °C, and humidity between 40% and 70%. Both sexes were utilised in all the experiments to mitigate the risk of bias. All animal procedures conformed to the UK legislation, Animals (Scientific Procedures) Act 1986 (United Kingdom), and the University of Oxford Ethical Review Committee policy, with a final ethical review by the Animals in Science Regulation Unit (ASRU) of the UK Home Office.

Immunohistochemistry

Mice, 8 to 14 weeks old of both sexes, were anaesthetised using pentobarbital (200 mg/mL) intraperitoneally, and transcardially perfused using PBS (0.1M, 20 mL), followed by ice-cold 4% PFA (20 mL). The brain was then extracted and stored in 4% PFA for 3h at RT, followed by successive sucrose solutions (final concentration 30%) as cryoprotectants at 4˚C, embedded in OCT (Fisher Scientific, 23-730-571), and stored at -80 ˚C until use. Sections, 30 µm thick, were prepared using a Leica Cryostat (CM3050S).

Whole-mount lateral ventricle staining was performed as described previously by Mirzadeh Z. et al. (2010) [19]. The sections were then washed in PBS for 10 min thrice and blocked with 5% fish gelatin and 0.5% Triton X in PBS for one hr at RT. The sections were then incubated overnight at 4˚C with primary antibodies diluted in blocking buffer (Table S3). The sections were then washed thrice with 0.1% Triton X in PBS for 10 min and incubated with the secondary antibody diluted in blocking buffer (1:1000, Table S1) for two hrs at RT. The sections were then DAPI-stained (1:10000 in PBS) for 30 min at RT and washed thrice in PBS for 10 min. The sections were dried and mounted on slides using VectaShield (Vector Laboratories, H-1000), then covered with a coverslip and sealed with nail varnish.

Imaging

Immunostained sections were then imaged using an upright widefield epifluorescence microscope (Leica, DM6000B), and images were acquired with a monochrome camera (DFC365FX). For confocal imaging, a Zeiss 780 upright LSM confocal microscope with an MP laser was used. The following objectives were employed for image acquisition: HCX PL APO

10x/0.40 CS, HC PL 20x/0.75 CS2, and HCX PL APO 40x/0.85 CORR CS. Image analysis was carried out using ImageJ Fiji (v1.54p).

Cell Counting

For the stereological analysis to quantify tdTomato^{+} cells in the V-SVZ and their colocalisation with markers of the V-SVZ neurogenic niche, three *PenkCre^{Ai9}* mouse brains were used in this study. Sequential coronal sections were obtained using the cryostat, each 30μm thick and spanning the entire striatum (3775 μm). Every 21st section was selected for antibody staining and cell counting, resulting in six coronal sections analysed for each quantification. According to the Allen Mouse Brain Atlas, sections spanned from Bregma Level: 1270 μm to Level: -1730 μm. DAPI was used to identify the V-SVZ on the lateral walls of the lateral ventricles. The total number of tdTomato^{+} cells, including those colocalised with different antibodies, was counted manually. The total number of cells across the striatum was calculated using the following formula: $Total\ number\ of\ cells\ across\ the\ striatum = l\ /(C \times n\ )\times N.$
Where N is the total number of cells counted in all sections of each mouse, l is the length of the striatum (3775 μm), C is the cross-sectional width of each section used (30μm), and n is the number of sections used (6) in the analysis.

For the 5-FU experiment, 12 (5-week-old) *PenkCre^{Ai9}* mice (6 males and 6 females) were used, divided into the 'Control' and 'Treated' groups (N=3 per group). Mice were treated with either saline (controls) or 250 mg/kg of 5-FU (Treated) with a single intraperitoneal injection. Twenty-four hours after the treatment, the mice were sacrificed, perfused, and the brains were harvested. Perfused brain samples were fixed overnight at 4°C in 4% paraformaldehyde, flash frozen and embedded in OCT and stored at -80°C till further use. Sequential coronal sections of thickness 30μm were obtained on a Leica Cryostat spanning the striatum from Bregma 1.42mm to Bregma -0.08 mm (Allen Mouse Brain Atlas). One in every 12 sections and four sections per animal were selected for immunostaining and cell counting in this experiment. For Ki67 and GFAP, the total number of cells across the striatum was calculated using the above formula: $Total\ number\ of\ cells\ across\ the\ striatum = l\ /\ (C \times n) \times N.$ Where N is the total number of cells counted in all sections of each mouse, l is the length of the striatum used in analysis (1200μm), C is the cross-sectional width of each section used (30μm), and n is the number of sections used (4) in the analysis. For KiR7.1, the counting method is explained in detail below.

Manual Quantification of Cell Populations and Colocalization of KiR7.1 and tdTomato^{+} cells

Single-cell quantification and colocalization analysis were performed using the open-source bioimage analysis software QuPath (v0.6.0). All images were acquired using a Leica DM6000B widefield fluorescence microscope equipped with a 20× objective (HC PL APO 20×/0.70 DRY) and a Leica DFC365FX camera at identical acquisition parameters across all animals (ALEXA 488: exposure 0.4s, gain 4, light intensity 5%). Multi-channel images were evaluated at 1:1 pixel resolution (100% zoom) with brightness and contrast settings optimised independently for

each fluorophore for visualisation purposes only; these display adjustments did not alter the underlying pixel data used for quantification. Cells within the V-SVZ were manually enumerated by an experienced researcher blinded to experimental conditions using the point annotation function of QuPath. Distinct object classes were defined to capture single and double-positive populations: tdT+ (red signal only) and tdT+/KiR7.1+ (colocalised; defined by strict spatial overlap of both fluorophores at the single-cell level). Positive cellular identification required distinct fluorescence exceeding local background thresholds. To ensure rigorous spatial consistency, tissue sections were strictly anatomically matched between treated and untreated animals across three distinct rostro-caudal levels. Cell counts were performed systematically through each anatomical region of interest by assigning a single point annotation to each positively identified cell. To correct for variations in the anatomical size of the SVZ across sections and animals, absolute point counts for each phenotypic class were normalized to the geometric area of their respective manually drawn ROI annotation, yielding standardized cellular densities expressed as cells/mm^2. Since the rodent V-SVZ is a functionally symmetrical structure at baseline, cellular densities from both hemispheres were averaged where available and of sufficient structural integrity to yield a single value per animal per section level. Where only one hemisphere was available due to tissue loss or sectioning artefact, the unilateral density was used directly. Finally, section-level densities across up to three rostro-caudal levels were averaged to generate a single representative value per animal for downstream statistical analysis.

Quantification of KiR7.1 Fluorescence Intensity

Fluorescence intensity of KiR7.1 was quantified using QuPath (Version, 0.6.0). To ensure valid semi-quantitative comparisons across all cohorts, all tissue sections were imaged under identical acquisition parameters (20x objective, 0.46 µm/px spatial resolution, 0.4s exposure, gain 4, 5% light intensity) to maintain a consistent 8-bit dynamic range. Because KiR7.1 exhibits a diffuse staining pattern that precludes accurate single-cell segmentation, an annotation-based regional measurement approach was utilised. For each image, a region of interest (ROI) was manually annotated to tightly encompass the target tissue structure, and the Mean Fluorescence Intensity (MFI) of the KiR7.1 channel was extracted. To account for non-specific tissue autofluorescence and baseline camera noise, a secondary background ROI was drawn in an adjacent, non-fluorescent region of the same image. The MFI of the background region was subtracted from the tissue ROI MFI to yield the final background-corrected mean intensity.

FACS sorting procedure

Mice were anaesthetised with pentobarbital and perfused with artificial cerebrospinal fluid in sucrose. To isolate neural stem/progenitor cells (NSCs/NPCs) from the V-SVZ of the lateral ventricles, microdissection of the lateral walls of the lateral ventricles was performed, as shown in Fig. S4a, which includes some surrounding striatal parenchyma. Tissue was chopped and placed in Accutase (Stem Cell Technologies, 07920) for 30 min at 4˚C, centrifuged for 2 min at 450 g, and the pellet was resuspended in Hibernate A (Brain Bits, HA). The pellet was triturated

and crudely filtered through 100- and 40-µm cell strainers. A Percoll (Sigma, P1644) density gradient was set up, and the cell suspension was placed on the top layer, followed by centrifugation at 430 g for 5 min at 4˚C. The top layer containing debris was aspirated, and the remaining layers were mixed and returned to the centrifuge for 5 min at 550 g. The pellet was resuspended in Hibernate A. Cells were counted using LIVE/DEAD viability dye (Thermo Fisher, L34963). The dye was removed by centrifuging the cells for 5 min at 550 g, followed by resuspension in Hibernate A. Cells were then either sorted directly as tdTomato+ (dataset B) or first incubated with CD11b_APC antibody (1:400, BioLegend, 101211) for 15 min, to eliminate a microglia signature (dataset A), followed by centrifugation for 5 min at 550 g, and resuspension in Hibernate A. The cells were then sorted as tdTomato$^+$/CD11b$^-$ population (dataset A) using the FACSAria II Cell Sorter (BD Biosciences) with a 100 µm nozzle, as previously described[37]. An example of the gating strategy used is shown in Fig S13.

Smart-seq2

We generated two single-cell RNA-seq datasets. Dataset A included cells from mice aged 9 weeks (three), and dataset B included cells from male mice aged 12 weeks (three). The main difference between the datasets was the FACS strategy employed. In dataset A, we collected the tdTomato$^+$/CD11b$^-$ population, whereas in dataset B, we collected all tdTomato$^+$ cells. Single cells processed by FACS were collected into 96-well plates (Thermo Fisher, AB0800) containing 0.4% Triton + RNase inhibitor (2 µL); dNTPs (10mM; 1µL); Oligo dT$_{30VN}$ (10 µM; 1 µL). The plates were then spun down at 700 g, frozen on dry ice, and stored in -80 ˚C until further processing. Reverse transcription was performed using Superscript II first strand buffer (5x, 2 µL); DTT (100 mM, 0.5 µL); Betaine (5M, 2 µL); MgCl2 (1M, 0.1 µL); RNase inhibitor (40 U/µL, 0.25 µL); TSO (100 µM, 0.1 µL); Superscript II RT (200 U/µL, 0.25 µL); and water (0.8 µL). Then, the cDNA was preamplified using KAPA HiFi HS Ready mix (2x, KAPA Biosystems, KK2601); ISPCR primers (10 µM) and water as per manufacturer instructions. The cDNA was purified using Agincourt Ampure XP beads (Beckman Coulter, A63881). The cDNA-bound beads were washed with 80% ethanol, and the pellet was resuspended in elution buffer (Qiagen, 19086). The beads were then removed by placing the mixture on the magnetic stand, and the eluted cDNA was placed into fresh 96-well plates. The cDNA quality check was performed using a Bioanalyzer according to the manufacturer's instructions (Agilent Technologies).

Cells were then tagmented and indexed using the Nexetra XT kit (Illumina, FC-131-1096). Briefly, Tagmentation Buffer (2.5 µL), cDNA (1.25 µL), and Amplicon Tagment Mix (1.25 µL) were added to each well. The plates were sealed, vortexed, and spun down, then tagmented at 55 ˚C for 5 min. The tagmentation was stopped by Neutralisation buffer (1.25 µL). The indexing was performed by adding Nextera PCR master mix (3.75 µL), Index 1 primers (1.25 µL), and Index 2 primers (1.25 µL) per well, followed by PCR as described previously[53]. The DNA concentration was quantified using the Quant-It PicoGreen system (Thermo Fisher, P7589) according to the manufacturer's instructions. The samples were denatured with NaOH (0.2 M, 5 µL) for 5 min and quenched with Tris-HCl (200 mM). The library was diluted to 1.8 pM in

buffer HT1, then loaded onto the chip and sequenced using the NextSeq® 500/550 High Output Kit v2 (75 cycles; Illumina TG-160-2005). Single-end sequencing was performed, and the FASTQ files were generated.

Single-cell RNA-seq data analysis

Preprocessing, quality control and filtering

First, raw FASTQ files generated per cell were concatenated, followed by FASTQC (v0.12.1) quality check. Then, we aligned the reads to the GENCODE mouse genome release M37 (GRCm39) using STAR (v2.7.10b)[45]. Reads were counted using the featureCounts function from the subread package (v2.1.1)[46] using the GENCODE vM37 primary assembly annotation. The resulting counts file was then imported into R and processed using Seurat (v5.2.0)[17]. We first removed genes expressed in fewer than 5 cells, and genes without annotations, and then removed cells with mitochondrial reads > 10% [47], < 500,000 counts, and < 500 unique features. The resulting sequencing depths for the final cells that passed quality control in both datasets were as follows: dataset A: 2,114,222 reads/cell; dataset B: 2,313,510 reads/cell.

Summary of sequenced and cell composition for Dataset A and Dataset B.

Dataset A, we initially sorted 260 cells across three plates (from 3 mice); however, due to technical issues during library preparation, two of the sorted plates yielded extremely low RNA/cDNA recovery and were therefore not processed for sequencing. As a result, only 76 cells from a third plate were ultimately sequenced and subjected to downstream QC analysis. The number of cells retained after QC and filtering was 41, of which B0 cells (N = 18), B1 cells (N = 6), and neuroblasts (NBs, N = 17). **Dataset B**, total cells sequenced 375 (from 3 mice), number of cells retained after QC and filtering 365, of which 41 had a neurogenic signature, B0 cells (N = 16); neuroblasts (NBs, N = 25). The remaining 324 cells exhibited a microglial signature and were discarded. While FACS is a powerful tool for isolating specific cell types from complex brain tissue, it imposes severe physical and physiological stresses to which adult neurons, unlike embryonic or NSCs in the V-SVZ, are particularly vulnerable. Thus, using the dissection and sorting protocol described here, we were unable to collect adult single neurons. However, we have previously successfully isolated adult brain bulk cells[37,35]. We believe that the dissection time and the procedure for dissociating adult brain tissue into a single-cell suspension, involving mechanical tearing and enzymatic digestion, followed by FACS for single-cell analysis, can compromise neuronal survival and integrity. This is supported by the literature[48].

Bioinformatics analysis

We then performed data normalisation, variable feature selection, data scaling, and principal component analysis using the standard Seurat functions. Dimension reduction was performed using RunUMAP function from Seurat. We then clustered cells using hierarchical clustering

with Ward's method and the anticorrelation matrix as a dissimilarity measure. We ran FindAllMarkers (MAST)[49] to identify cluster-specific markers and manually annotated the cell types based on the expression of top marker genes. Plotting was done using the scCustomize package (v3.0.1) and SCpubr. For differential expression between cell types, we used FindMarkers (DESeq2)[50] and applied the FDR method to adjust for multiple comparisons. We performed gene ontology enrichment analysis on genes with FDR < 0.05 separately for upregulated and downregulated genes using the enrichGO function from the clusterProfiler (v4.14.4)[51] package on all ontologies, with pAdjustMethod specified as "BH" and qvalueCutoff of 0.05. For data integration between dataset A and dataset B, we employed the standard Seurat pipeline, based on FindIntegrationAnchors, followed by principal component analysis and dimensionality reduction on the integrated assay, while retaining cluster labels from the original analyses. For Pseudotime analysis, we fit a Pseudotime trajectory using Slingshot (v2.14)[52] and scaled it from 0 to 100. We then used tradeSeq (v1.20)[53] to fit a generalised additive model to the normalised data along the Pseudotime trajectory to identify genes associated with Pseudotime (nknots = 3). We used the pheatmap (v1.0.12), tidyverse (v2.0.0), and tradeSeq's built-in plotting functions for data visualisation.

Analysis of scRNA-seq data from Dulken *et al.* (2017) study

We downloaded the metadata and counts matrix provided by Dulken *et al.*[20] for their final dataset. To identify genes that change significantly with Pseudotime in the Dulken dataset, we created a Seurat object while retaining the cell labels provided by the authors, preprocessed the data, and applied slingshot and tradeSeq as described above.

To integrate the Dulken dataset with our dataset, we first identified genes expressed in both datasets and filtered both count matrices to retain only the shared genes. We then merged the count matrices, imported them into Seurat, and retained the authors' original cell labels. Finally, we performed data normalisation, variable feature selection, data scaling, and principal component analysis using Seurat's default features. We then performed dimension reduction using the RunUMAP function on the first 10 principal components.

Analysis of single-cell RNA-seq data from the human subventricular zone

For the Puvogel *et al.* (2024)[36] dataset, we downloaded the count matrix and metadata file from GEO (GSE234790) and created a Seurat object retaining the authors' cluster labels. We then performed data normalisation, variable feature selection, data scaling, principal component analysis, and dimension reduction (dims 1:50) using standard Seurat functions. Then, we subset the dataset to include only the Astro/NSCs cluster and added a metadata column to classify cells as *KCNJ13*+ or *KCNJ13*- based on whether *KCNJ13* expression was > 0. We then performed differential expression between the two NSC subtypes using the FindMarkers function (MAST, latent.vars = sex, ethnicity, age, and PMI). Then, for genes with a p-value < 0.05, we performed gene ontology analysis using the clusterProfiler (v4.14.4) package on Biological Processes ontology, with pAdjustMethod specified as "BH" and pvalueCutoff set to 0.05, separately for upregulated and downregulated genes.

High-throughput microfluidic gene expression

The protocol used in this experiment has been previously described[38]. The mice used were 2 months of age. Briefly, mice were anaesthetised with pentobarbital and perfused with artificial cerebrospinal fluid in sucrose. To isolate neural stem/progenitor cells (NSCs/NPCs) from the V-SVZ of the lateral ventricles, microdissection of the lateral walls of the lateral ventricles was performed, as shown in Fig. S4a, which includes some surrounding striatal parenchyma. The protocol follows the steps outlined in the FACS sorting procedure above, with a few exceptions. Using the gating conditions shown in Fig. S11, cells were incubated either with KiR7.1 antibody (ATTO488 (Allomone) @ 1:50) and CD11b_APC antibody (1:400, BioLegend, 101211) for 15 min on ice and in the dark, or with the CD11b_APC antibody alone, before collecting 10 cells in triplicate from each population (KiR7.1$^+$/ tdTomato$^+$/CD11b$^-$ and KiR7.1$^-$/tdTomato$^+$/CD11b$^-$). Collected cells were then subjected to high-throughput microfluidic gene expression analysis, as previously described[38]. The following TaqMan assays were obtained from ThermoFisherScientific: *Kcnj13* (Mm01287179_m1); *Prlr* (Mm04336676_m1); *Enpp2* (Mm00516572_m1); *Penk* (Mm01212875_m1); *Sox11* (Mm01281943_s1); *Slc1a3* (Mm00600697_m1) and *Rn45s* (Mm04277571_s1). Data were analysed as described previously using the ΔΔCt method[54], and plotted using the pheatmap (v1.0.12) function from R.

6-OHDA stereotaxic injection

Mice, 12-14 weeks old, both males and females, were anaesthetised with isoflurane (0.5-3% oxygen gas mixture, 2 l/min) and given buprenorphine (0.1 mg/kg body weight) for peri-operative analgesia during stereotaxic injections. The injections targeted unilaterally to the striatum at two sites (rostral site: +0.6 mm anterior, +2.3 mm lateral from Bregma at a depth of -2.5 mm from the brain surface and caudal site: -0.1 mm posterior and +2.4 mm lateral to Bregma at a depth of -2.4 mm from the brain surface. Either 6-hydroxydopamine (6-OHDA [6 mg/ml] Sigma, Cat #: H4381-100MG) dissolved in saline and 0.02% ascorbic acid) or the vehicle alone (0.02% ascorbic acid in saline) were injected (750 nl per site) at a maximum rate of 100 nl/min using a glass micropipette lowered to the target site; the micropipette was left in place 5 minutes before and after the injection. Recovery of mice was monitored daily. 5-bromo-2'-deoxyuridine (BrdU) was obtained from Sigma (B5002). BrdU solution was prepared at 20 mg/kg in 0.9% NaCl and injected intraperitoneally at a final concentration of 200 mg/kg (equivalent to 10 µl/g).

Statistical analysis

Unpaired data were analysed using unpaired t-tests and met the assumptions of normality, homoskedasticity, and the absence of correlation in residuals. Paired data (for the 6-OHDA injection) were analysed using a paired t-test and were tested for assumptions as mentioned above. $P < 0.05$ was considered statistically significant throughout. Statistical tests and plotting were done using GraphdPad PRISM (v10.6.0).

Table S3: List of primary and secondary antibodies used in the study.

Primary Antibody	Host Species	Supplier	Cat. No.	Dilution
BRDU	Rat	Novus	NB500-169	1:200
CALBINDIN	Mouse	Sigma	CB-955	1:400
CALRETININ	Rabbit	Swant	7699/4	1:1000
CD11B-APC	Mouse	BioLegend	101211	1:400
CD133	Rat	Ebioscience	14-1331-80	1:500
CD31	Rat	Ebioscience	MEC 13.3	1:100
DCX	Rabbit	Abcam	ab18723	1:200
GFAP	Rabbit	Dako	GA524	1:500
GLAST	Guinea Pig	Merck Millipore	AB1783	1:500
KCNJ13/KIR7.1* (IF)	Mouse	Santa Cruz	sc-398810	1:100
KI67	Rabbit	Abcam	ab16667	1:5000
KIR7.1-ATTO488* (FACS)	Rabbit	Alomone Labs	APC-125-AG	1:50
PARVALBUMIN	Rabbit	Swant	PV-25	1:1000
PENK	Rabbit	Neuromics	RA14124	1:1000
PRLR	Mouse	Novus	NB300-561AF488	1:100
SOX11	Guinea Pig	Dr. Elisabeth Sock	—	1:2000
TH	Mouse	Millipore	Mab318	1:1000

Secondary Antibody	Supplier	Cat. No.	Dilution
Alexa Fluor 488 Goat anti-Guinea Pig IgG (H+L)	Invitrogen (Thermo Fisher)	A-11073	1:1000
Alexa Fluor 488 Goat Anti-Rat IgG (H+L)	Invitrogen (Thermo Fisher)	A-11006	1:1000
Alexa Fluor 488 Goat Anti-Mouse IgG (H+L)	Invitrogen (Thermo Fisher)	A-11029	1:1000
Alexa Fluor 555 Donkey Anti-Mouse IgG (H+L)	Invitrogen (Thermo Fisher)	A-31570	1:1000
Alexa Fluor 555 Donkey Anti-Rabbit IgG (H+L)	Invitrogen (Thermo Fisher)	A-31572	1:1000
Alexa Fluor 647 AffiniPure Donkey Anti-Guinea Pig IgG (H+L)	Jackson ImmunoResearch	706-605-148	1:1000
Alexa Fluor 647 AffiniPure Donkey Anti-Rat IgG (H+L)	Jackson ImmunoResearch	712-605-153	1:1000
Alexa Fluor 647 Donkey Anti-Mouse IgG (H+L)	Invitrogen (Thermo Fisher)	A-31571	1:1000
Alexa Fluor 647 Donkey Anti-Rabbit IgG (H+L)	Invitrogen (Thermo Fisher)	A-31573	1:1000

Additional references

1 Doetsch, F., Caille, I., Lim, D. A., Garcia-Verdugo, J. M. & Alvarez-Buylla, A. *Cell* **97**, 703–716 (1999).
2 Scholzen, T. & Gerdes, J. *J Cell Physiol* **182**, 311–322 (2000).
3 Bergsland, M., Werme, M., Malewicz, M., Perlmann, T. & Muhr, J. *Genes Dev* **20**, 3475–3486 (2006).
4 Chaker, Z., Codega, P. & Doetsch, F. *Wiley Interdiscip Rev Dev Biol* 5, 640–658 (2016).
5 Besusso, D. *et al. Nat Commun* **4**, 2031 (2013).
6 Fuentealba, L. C. *et al. Cell* **161**, 1644–1655 (2015).
7 Alvarez-Buylla, A. & Garcia-Verdugo, J. M. *J Neurosci* **22**, 629–634 (2002).
8 Yang, J. *et al. Front Neuroanat* **10**, 72 (2016).
9 Morsch, M. *et al. Front Cell Neurosci* **9**, 321 (2015).
10 Dissing-Olesen, L. *et al. Nat Commun* **14**, 6015 (2023).
11 Garcia, J. A., Cardona, S. M. & Cardona, A. E. *Curr Protoc Immunol* **104**, 14 35 11–14 35 15 (2014).
12 Zhang, X., Goncalves, R. & Mosser, D. M. *Curr Protoc Immunol* **Chapter 14**, 14 11 11–14 11 14 (2008).
13 Kim, B., Kim, T., Im, H., Shin, K. S. & Kang, S. J. *Biochem Biophys Res Commun* **742**, 151157 (2025).
14 Richardson, S. J., Lemkine, G. F., Alfama, G., Hassani, Z. & Demeneix, B. A. *Neurosci Lett* **421**, 234–238 (2007).
15 Bond, A. M., Bhalala, O. G. & Kessler, J. A. *Dev Neurobiol* **72**, 1068–1084 (2012).
16 Orsolits, B. *et al. Stem Cells Dev* **22**, 2777–2793 (2013).
17 Hao, Y. *et al. Nat Biotechnol* **42,** 293–304 (2024).
18 Jiao, X. *et al. Front Cell Dev Biol* **9**, 810020 (2021).
19 Mirzadeh, Z., Doetsch, F., Sawamoto, K., Wichterle, H. & Alvarez-Buylla, A. *J Vis Exp* (2010).
20 Dulken, B. W., Leeman, D. S., Boutet, S. C., Hebestreit, K. & Brunet, A. *Cell Rep* **18**, 777–790 (2017).
21 Negishi, K. *Neurosci Res* **19**, 21–29 (1994).
22 Arvidsson, A., Collin, T., Kirik, D., Kokaia, Z. & Lindvall, O. *Nat Med* **8**, 963–970 (2002).
23 Yamashita, T. *et al. J Neurosci* **26**, 6627–6636 (2006).
24 Llorens-Bobadilla, E. et al. *Cell Stem Cell* **17**, 329–340 (2015).
25 Grant, R. J. & Clarke, P. B. *Neuroscience* **115**, 1281–1294 (2002).
26 Wojtowicz, J. M. & Kee, N. *Nat Protoc* **1**, 1399–1405 (2006).
27 Lyu, Y. et al. *CNS Neurosci Ther* **27**, 1289–1299 (2021).
28 Tong, C. K. *et al. Cell Stem Cell* **14**, 500–511 (2014).
29 Obernier, K. & Alvarez-Buylla, A. *Development* **146** (2019).
30 Wang, W. *et al. J Biol Chem* **288**, 2623–2631 (2013).
31 Shingo, T. *et al. Science* **299**, 117–120 (2003).
32 Mak, G. K. *et al. Nat Neurosci* **10**, 1003–1011 (2007).
33 Vancamp, P. *et al. Sci Rep* **9**, 19689 (2019).
34 Audesse, A. J. & Webb, A. E. *Mech Ageing Dev* **191**, 111323 (2020).
35 Malik, M. Y. et al. *Nat Metab* **6,** 2100–2117 (2024).
36 Puvogel, S. et al. *eNeuro* **11** (2024).
37 Vatanashevanopakorn, C. *et al. Neuromethods* **143** (2017).

38 Nath, A. R., Drissen, R., Guo, F., Nerlov, C., Minichiello, *L. Neuromethods* **143** (2017).
39 Merkle, F. T., Tramontin, A. D., Garcia-Verdugo, J. M. & Alvarez-Buylla, A. *Proc Natl Acad Sci U S A* **101**, 17528–17532 (2004).
40 Anderson, A. G., Kulkarni, A. & Konopka, G. *Sci Rep 13*, 9031 (2023).
41 Chen, Y. J. et al. *Sci Rep* **7**, 45656 (2017).
42 Blackiston, D. J., McLaughlin, K. A. & Levin, M. *Cell Cycle* **8**, 3527–3536 (2009).
43 Bjorkgren, I. *et al. J Gen Physiol* **153** (2021).
44 Madisen, L. *et al. Nat Neurosci 13*, 133–140 (2010).
45 Dobin, A. *et al. Bioinformatics* **29**, 15–21 (2013).
46 Liao, Y., Smyth, G. K. & Shi, W. *Bioinformatics* **30**, 923–930 (2014).
47 Scandella, V., Petrelli, F., Moore, D. L., Braun, S. M. G. & Knobloch, M. *Trends Endocrinol Metab* **34**, 446–461 (2023).
48 Lacar, B. *et al. Nat Commun* **7**, 11022 (2016).
49 Finak, G. *et al. Genome Biol* **16**, 278 (2015).
50 Love, M. I., Huber, W. & Anders, S. *Genome Biol* **15**, 550 (2014).
51 Wu, T. et al. *Innovation (Camb)* **2**, 100141 (2021).
52 Street, K. *et al. BMC Genomics* **19**, 477 (2018).
53 Van den Berge, K. *et al. Nat Commun* **11**, 1201 (2020).
54 Drissen, R. *et al. Nat Immunol* **17**, 666–676 (2016).
